# Virulence Evolution of the Human Pathogen *Neisseria meningitidis* by Recombination in the Core and Accessory Genome

**DOI:** 10.1371/journal.pone.0018441

**Published:** 2011-04-26

**Authors:** Biju Joseph, Roland F. Schwarz, Burkhard Linke, Jochen Blom, Anke Becker, Heike Claus, Alexander Goesmann, Matthias Frosch, Tobias Müller, Ulrich Vogel, Christoph Schoen

**Affiliations:** 1 Institute for Hygiene and Microbiology, University of Würzburg, Würzburg, Germany; 2 Cancer Research UK Cambridge Research Institute, Li Ka Shing Centre, Cambridge, United Kingdom; 3 Center for Biotechnology, Bielefeld University, Bielefeld, Germany; 4 Faculty of Biology, University of Freiburg, Freiburg, Germany; 5 National Reference Laboratory for Meningococci (NRZM), Institute for Hygiene and Microbiology, University of Würzburg, Würzburg, Germany; 6 Department of Bioinformatics, Biocenter, University of Würzburg, Würzburg, Germany; University of Hyderabad, India

## Abstract

**Background:**

*Neisseria meningitidis* is a naturally transformable, facultative pathogen colonizing the human nasopharynx. Here, we analyze on a genome-wide level the impact of recombination on gene-complement diversity and virulence evolution in *N. meningitidis*. We combined comparative genome hybridization using microarrays (mCGH) and multilocus sequence typing (MLST) of 29 meningococcal isolates with computational comparison of a subset of seven meningococcal genome sequences.

**Principal Findings:**

We found that lateral gene transfer of minimal mobile elements as well as prophages are major forces shaping meningococcal population structure. Extensive gene content comparison revealed novel associations of virulence with genetic elements besides the recently discovered meningococcal disease associated (MDA) island. In particular, we identified an association of virulence with a recently described canonical genomic island termed IHT-E and a differential distribution of genes encoding RTX toxin- and two-partner secretion systems among hyperinvasive and non-hyperinvasive lineages. By computationally screening also the core genome for signs of recombination, we provided evidence that about 40% of the meningococcal core genes are affected by recombination primarily within metabolic genes as well as genes involved in DNA replication and repair. By comparison with the results of previous mCGH studies, our data indicated that genetic structuring as revealed by mCGH is stable over time and highly similar for isolates from different geographic origins.

**Conclusions:**

Recombination comprising lateral transfer of entire genes as well as homologous intragenic recombination has a profound impact on meningococcal population structure and genome composition. Our data support the hypothesis that meningococcal virulence is polygenic in nature and that differences in metabolism might contribute to virulence.

## Introduction

*Neisseria meningitidis* is a commensal exclusively of the human nasopharynx that is carried by about 20% of the human population [1], [2]. For reasons that are still largely unknown, the meningococcus can sometimes invade the pharyngeal mucosal epithelium causing septicemia or acute bacterial meningitis [3]. Based on the chemical composition and the immunological characteristics of their capsular polysaccharide, meningococci are divided into 12 serogroups [4], and the most important serogroups associated with disease in humans are A, B, C, W-135 and Y. While serogroups B and C cause the majority of cases in industrialized countries, serogroup A strains are the main meningococcal pathogens in sub-Saharan Africa [5]. Apart from epidemic outbreaks, approximately 500,000 cases of meningococcal disease are estimated to occur every year on a worldwide basis posing a heavy burden on the public health systems especially in developing countries [6].

The genetic basis for meningococcal virulence is still not fully resolved. Epidemiologic and experimental observations suggest that the polysaccharide capsule is necessary but not sufficient to confer full virulence. Unfortunately, the strict tropism of *N. meningitidis* for humans has so far prevented the development of any suitable animal model to study meningococcal infection biology in vivo. Analysis of meningococcal population structure by MLST suggests that disease-causing meningococci belong to particular groups of related sequence types (STs), termed clonal complexes (CCs), that are overrepresented in disease isolates relative to their carriage prevalences, and that only few so called hyperinvasive lineages are responsible for most disease (reviewed in ref. [7]). However, whole genome sequencing (WGS) as well as whole-genome mCGH demonstrated that there is no virulence gene pool in *N. meningitidis* that is exclusively present in all strains from hyperinvasive lineages (reviewed in ref. [8]), although one genome-wide association study (GWAS) found an association between the MDA island belonging to the M13 family of filamentous prophages with disease-causing bacteria from hyperinvasive lineages [9]. However, this GWAS included only genes from a meningococcal serogroup A strain and thus missed all genes that are specific for the genetically variant serogroup B and C strains causing most disease in industrialized countries.

Compared to the ongoing efforts in identifying meningococcal virulence-associated genes, more significant progress has been made in the development of molecular typing methods and MLST has emerged as a genotyping “gold standard” for *N. meningitidis* (reviewed in ref. [10]). It benefits from a well-established population genetic framework and has successfully been applied to the study of meningococcal diversity in the context of epidemiology and surveillance [7], [11]. However, since this method is based on the analysis of DNA sequence polymorphisms in ≈450-bp internal fragments of only seven housekeeping genes, strain relatedness is inferred from a very limited sub-sampling of the entire genome. This becomes increasingly relevant given the extensive genomic diversity observed in *N. meningitidis* through WGS [8], [12], [13], [14] and mCGH [15], [16], [17], [18]. In fact, *N. meningitidis* is a naturally transformable species and constitutively competent for the uptake of DNA and WGS revealed the presence of about 2000 copies of the 10-bp DNA uptake sequence (DUS) in meningococcal genomes facilitating the incorporation also of foreign DNA. Accordingly, instances of lateral gene transfer (LGT) and gene conversion involving the nonreciprocal replacement or addition of either homologous or non-homologous sequences, respectively, have been described in *N. meningitidis* (reviewed in [19]).

Little is known so far about the correlation between population structure inferred from mCGH and MLST data for bacteria undergoing frequent (homologous) recombination such as *N. meningitidis*. Likewise, a systematic genome-wide analysis of the extent to which genes from the meningococcal core genome are affected by recombination is still lacking.

Here, we used a combination of mCGH and computational approaches to systematically analyze recombination-mediated variation of the meningococcal core and accessory genomes in a set of 29 meningococcal strains from 22 different CCs. In particular, we assessed the correlation between meningococcal population structure based on the accessory and core genomes as revealed by mCGH and MLST analyses, respectively. We further analyzed gene content variation among strains to estimate the impact of recombination on the accessory genome with a particular focus on possible associations between gene content and pathogenicity. To systematically estimate the extent to which the core genome is affected by homologous (intragenic) recombination, we screened the core genome in a subset of strains from the same population for recombinant genes and performed a functional classification of all genes affected by recombination.

## Results

In this work, we analyzed the composition of the meningococcal gene complement of a population of 29 strains via mCGH. We used an oligonucleotide-based microarray which covers the genomes from the meningococcal strains α14, FAM18, MC58 and Z2491 and which allows for the simultaneous detection of 1679 genes at an overall accuracy of 98% [20]. To analyze the impact of recombination on genes from the core genome, we in addition computationally screened 1092 core genes in seven strains from the same population for signs of recombination.

### Characterization of the sample population

Since, by definition, a population is all the organisms that both belong to the same species and live in the same geographical area [21], we analyzed a national, well characterized collection of strains that was sampled predominantly in Germany in the years 1999–2000 to also avoid potential confounding effects of spatial and temporal population structure [22]. The sample population covered over 98% of the observed genetic diversity in a population of carriage strains with assigned CCs [1] and included all major hyperinvasive lineages associated with invasive disease as determined via MLST. The panel comprises 29 strains isolated from patients (n = 13) as well as from healthy carriers (n = 16) which belong to six different serogroups including also capsule null locus (*cnl*) strains and 22 different CCs, respectively (Table 1). Fifteen strains were from CCs more frequently associated with invasive meningococcal disease (IMD) than carriage, and 14 in turn belonged to CCs that are mostly associated with asymptomatic carriage in healthy individuals. Twenty-six strains (90%) were isolated in Germany either in the course of the Bavarian carriage study [1] or were taken from the strain collection of the German Reference Laboratory for Meningococci (NRZM, Würzburg, Germany) spanning the same time period. Strains Z2491, MC58 and FAM18 were included from outside Germany to allow for computational comparisons based on available whole genome sequences.

**Table 1:** Strains used in the study.

Strain	Serogroup	ST	CC	Lineage(1)	Source	Country	Year	Reference	Genome(2)
α14	*cnl*	53	53	carriage	carrier	Germany	1999	[1]	AM889136*
α704	*cnl*	198	198	carriage	carrier	Germany	2000	[1]	NA
WUE2594	A	5	5	invasive	IMD(3)	Germany	1991	[80]	NA
Z2491	A	4	4	invasive	IMD	Gambia	1983	[81]	AL157959*
DE6894	B	32	32	invasive	IMD	Germany	2000	[11]	NA
α4	B	19	18	carriage	carrier	Germany	1999	[1]	NA
α31	B	797	269	invasive	carrier	Germany	1999	[1]	NA
α78	B	44	41/44	invasive	carrier	Germany	1999	[1]	NA
α490	B	364	364	carriage	carrier	Germany	2000	[1]	NA
α522	B	35	35	carriage	carrier	Germany	2000	[1]	NA
α547	B	41	41/44	invasive	carrier	Germany	2000	[1]	NA
α710	B	136	41/44	invasive	carrier	Germany	2000	[1]	CP001561
DE7127	B	41	41/44	invasive	IMD	Germany	2000	[11]	NA
DE7865	B	269	269	invasive	IMD	Germany	2001	[11]	NA
DE7901	B	18	18	carriage	IMD	Germany	2001	[11]	NA
DE8638	B	162	162	carriage	IMD	Germany	2002	[11]	NA
DE9155	B	44	41/44	invasive	IMD	Germany	2002	[11]	NA
MC58	B	74	32	invasive	IMD	UK	1983	[82]	AE002098*
DE6904	C	8	8	invasive	IMD	Germany	2002	[11]	NA
DE7017	C	11	11	invasive	IMD	Germany	2000	[11]	NA
FAM18	C	11	11	invasive	IMD	USA	1980	[12]	AM421808*
WUE2121	C	11	11	invasive	IMD	Germany	1997	[83]	NA
α95	29E	106	106	carriage	carrier	Germany	1999	[1]	NA
α153	29E	60	60	carriage	carrier	Germany	1999	[1]	AM889137
α707	29E	254	254	carriage	carrier	Germany	2000	[1]	NA
α3	W-135	174	174	carriage	carrier	Germany	1999	[1]	NA
α275	W-135	22	22	carriage	carrier	Germany	2000	[1]	AM889138
α267	Y	23	23	carriage	carrier	Germany	2000	[1]	NA
α533	Y	92	92	carriage	carrier	Germany	2000	[1]	NA

(1) Assignments of clonal complexes (CCs) into invasive and carriage lineages is based on refs. [15].

(2) Asterisks behind the GenBank accession numbers indicate that the respective genome was represented on the microarray used for mCGH analyses.

(3) Invasive meningococcal disease.

Based on the concatenated sequences of the seven MLST housekeeping gene fragments comprising the entire set of 29 strains, Tajima's D was slightly but non-significantly negative (D = −1.52788, p>0.10) implying that the population might have experienced (small) changes in population size such as population bottlenecks. There was also no significant subdivision within the German population (p>0.10), and there were also no genetic differences detectable between the two meningococcal subpopulations isolated from patients and healthy carriers, respectively. However, hyperinvasive and carriage populations were genetically distinct (p<0.05) as revealed by the K_s_*, Z* as well as S_nn_ statistics, and the subpopulation of carriage strains showed a higher pairwise nucleotide diversity (π = 0.04498) than strains belonging to hyperinvasive lineages (π = 0.003104). Based on the individual MLST loci, there were signs of recombination in the allele sequences of *abcZ*, *aroE*, *pdhC* and *pgm*, and the mean relative recombination to mutation rate ρ/θ was estimated to be about 1.25 (CI_0.95_ = [0.114, 2.851]) which is comparable with earlier estimates based on larger sample sizes [23] (Table S1). Taken together, this sample therefore reflects some important genetic characteristics found in larger carriage and disease populations [7], [24].

### Characterization of the meningococcal gene pool

By definition, the bacterial pan-genome consists of the set of genes that can be found in all strains of that species, i. e., the species' core genome, and the accessory genome which is composed of genes that vary between strains [25]. Among the 1679 genes compared, 1139 were found in all of the 29 strains examined via mCGH and are thus likely part of the meningococcal core genome. Five hundred forty genes in turn were found to be absent in at least one strain of the sample collection and are thus part of the meningococcal accessory genome. Of these, 31 genes could only be found in one of the four genomes represented on the array but not in any other of the additional 25 strains examined. With respect to the sample population, they consequently constitute strain-specific genes. The remaining 509 accessory genes are present in more than one meningococcal strain and are thus part of the meningococcal distributed genome.

**Figure 1:**
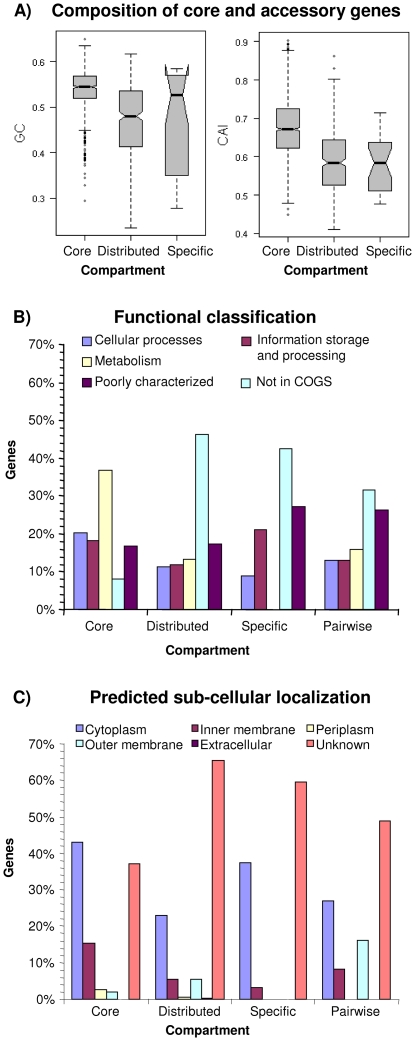
Characterization of the meningococcal gene complement. A) Boxplots comparing the GC content and CAI between core, distributed and strain-specific genes. Core genes have a significantly higher GC content than the distributed genes and also a significantly higher CAI than genes from the accessory genome comprising distributed and strain-specific genes (p<0.001, Wilcoxon test). B) Distribution of genes from the different genomic compartments also including pairwise differences between strains from the same CC among the major COG functional classes. There are significant differences in the functional composition between all four genomic compartments (p<0.01, χ^2^ test). Compared to the other genomic compartments the core genome is enriched for metabolic genes as well as genes involved in cellular processes, information storage and processing, respectively (p<0.01, Fisher's exact test). C) Histogram of the predicted subcellular localization of genes from the different genomic compartments. Again, there are significant differences between the different genomic compartments (p<0.01, χ^2^ test). In line with their housekeeping functions, core genes are localized primarily within the cytoplasm, the inner membrane and the periplasmic space whereas the distributed genes and genes that differ between strains from the same CC are in turn enriched for genes coding for outer membrane proteins (p<0.05, Fisher's exact test).

Compared to accessory genes, core genes have a higher GC content and codon adaptation index (CAI) (Figure 1 A), and the distribution of core, distributed and strain-specific genes among the different functional classes are also significantly different (Figure 1 B). The core genome is enriched for genes in housekeeping functions and in particular for metabolic genes which are entirely missing among the strain-specific genes. In line with their housekeeping functions, core genes encode proteins predominantly localized in the cytoplasm, inner membrane or periplasmic space while the distributed genome is enriched for genes encoding outer membrane proteins (Figure 1 C). The latter includes proteins involved in host cell interactions such as the major adhesin OpcA (NMB1053), the autotransporter proteins NalP (NMB1969), NadA (NMB1994) and MspA (NMB1998), as well as the two-partner secretion (TPS) proteins TpsA2 (NMB1768) and TpsA3 (NMB1214) (see below), and it is likely that the host immune system exerts positive selection pressure which favors a high variability in the adhesin repertoire. Compared to the distributed genome, the strain-specific genes are slightly enriched for genes involved in information storage and processing, and their functional profile together with the low GC content suggest that they might reside on mobile genetic elements (MGEs). In fact, of the 31 strain-specific genes 16 belong to a Mu-like prophage found in the α14 genome [14], and another four genes are also located on different Mu-like prophages in the genomes of the strains α14 and Z2491, respectively. Another ten genes reside on (candidate) minimal mobile elements ((c)MMEs). This class of MGEs has been defined as regions encompassing two conserved genes between which different whole-gene cassettes are found in different strains and that are chromosomally incorporated through the action of homologous recombination [26]. Finally, NMB0374 encodes a MafB1 protein with a highly variant C-terminus so that the respective oligonucleotide probably does not hybridize with any other C-terminal cassette.

### Meningococcal population structure based on gene content

**Figure 2:**
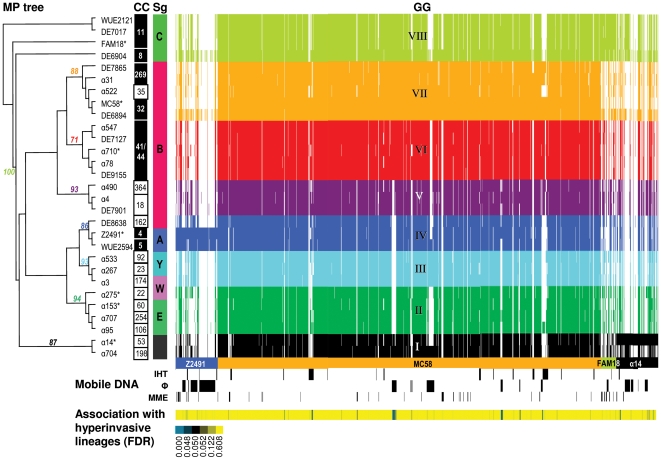
Clustering of strains based on the accessory genome. A maximum parsimony (MP) tree is shown with bootstrap values at nodes used for grouping of strains into eight GGs from their gene content comprising 470 parsimony informative genes as revealed by mCGH. Strains with an asterisk next to their name have further been used for the in silico screening for intragenomic recombination in 1092 genes from the core genome as estimated via mCGH of the entire sample population. Next to the MP tree, the CCs and serogroups (Sg) of the respective strains are given with hypervirulent CCs in black boxes, and right to the Sgs a virtual array image displaying the presence and absence of 1679 genes is shown. Strains from the same serogroup have in general highly similar gene content, and strains from the same CC always belong to the same GG. In turn, a GG can comprise strains from different CCs, and with the exception of the two serogroup W-135 strains split between GG-II and GG-III and the serogroup B strain DE8638, GGs always comprise strains from the same serogroup. However, no two strains have exactly the same gene content (see also Figure 1 for the functional profile of genes differently present among otherwise identical pairs of strains). Right below the virtual array, the spotted genes are color coded according to the source genome (representing the genomes of strain Z2491, MC58, FAM18 and α14), and the presence of putatively mobile DNA is depicted below with IHT-B, IHT-C, IHT-E as well as the λ prophage denoted as B, C, E and λ, respectively, in the respective lanes (Abbreviations: IHT, island of horizontal transfer; Φ: prophage; MME, minimal mobile element). At the lower margin, the FDR for the association with hyperinvasive lineages is color coded for each gene with genes having a FDR<0.05 depicted in blue.

**Figure 3:**
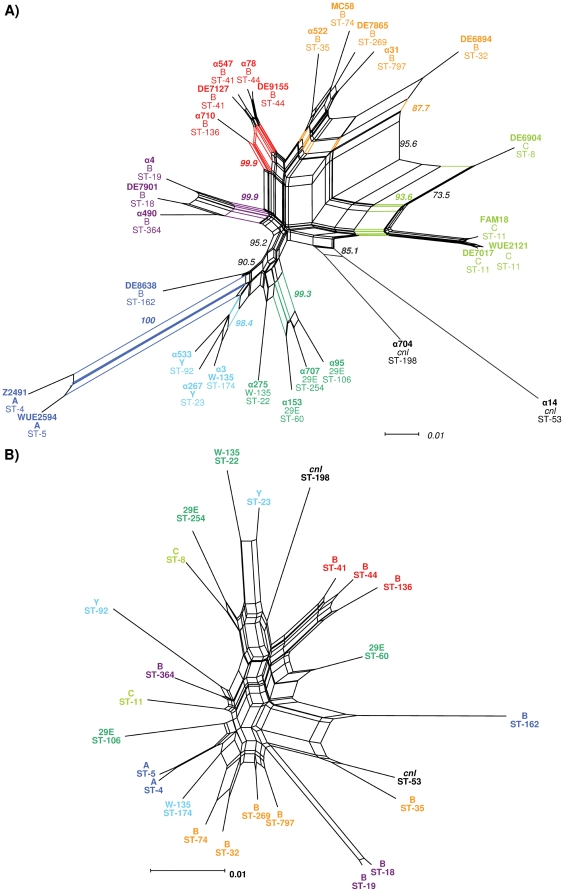
Comparison of strain clustering based on mCGH and MLST. A) Neighbor-net representation of gene distances based on the distribution of genes from the accessory genome. Strain names as well as the defining splits having a bootstrap support of greater than 85% are printed in the same colors as in Figure 2. Clustering based on gene content distance and maximum parsimony results in the same eight genome groups. Serogroup B and C strains as well as the two *cnl* strains are separated from the other four serogroups based on gene content, and the cycles within the serogroup B/C subgraph indicate a high rate of gene transfer among these strains. Strains from the same CC always reside on common splits. B) Neighbor-net representation based on the concatenated alignments of the seven housekeeping gene fragments form the meningococcal core genome used for MLST comprising 383 parsimony informative sites. A MLSA-based clustering of strains results in the separation of strains from the same serogroup or belonging to the same GGs. The large number of cycles in the graph indicates a high rate of homologous recombination among the genes used for MLST and that a MLSA-based reconstruction of the deep phylogeny of these strains is not possible.

Maximum parsimony (Figure 2) as well as distance based clustering methods (Figure 3 A) show that the sample population can be grouped into eight genome groups (GGs) based on gene content. Each GG contains between two and five strains and together the eight GGs form two larger groups. One of these two groups comprises GG-I and GG-V to GG-VIII and contains almost all serogroup B and C strains (17/18) as well as the two *cnl* strains, while the other group comprises GG-II, GG-III and GG-IV and contains almost exclusively non-B/C serogroup strains (9/10). All strains from GG-I and GG-V to GG-VIII harbor either a complete copy of a λ-like prophage or a degraded version thereof in form of a canonical genomic island termed IHT-E [16]. In addition, all strains from GG-V to GG-VII contain IHT-B and with the exception of strain α522 (ST-35 CC) also a complete IHT-C. IHT-C in turn is missing in all strains from GG-II, GG-III and GG-IV with the exception of strain α95 (ST-106 CC) in GG-II. However, not all GGs are separated from each other by the specific presence or absence of certain marker genes, and most genes specifically present or absent in certain GGs are located either at the *cps* locus or on a variety of MMEs (Table S2). For example, the two *cnl* strains form GG-I and they accordingly lack genes from the *cps* locus, and strains from GG-VII and GG-VIII harbor a number of MMEs that cannot be found in the other genome groups. Notably, no two strains have exactly the same gene content, and even strains from the same clonal complexes and the same serogroup differ particularly in their repertoire of (c)MMEs and neisserial filamentous prophages (Nfs) [27] (Table S3). These data suggest a high rate of LGT among strains primarily in the form of (c)MMEs and phages of the Nf family. In line with these observations, the high degree of homoplasy in the gene content data, the correspondingly low ensemble retention index (RI) of the maximum parsimony tree (Figure 2) with RI = 0.643 and the numerous cycles in the neighbor-net reconstruction (Figure 3 A) suggest a pervasive and genome-wide effect of LGT on meningococcal gene content evolution and population structure.

Despite the differences described above, strains belonging to the same serogroup have also a highly similar gene content (Figure 2 and 3 A). For example, all strains in GG-VIII belong to serogroup C, and all strains in GG-VI and GG-VII to serogroup B, respectively. Likewise, strains belonging to the same CC have highly similar gene content, and clustering of strains based on mCGH data reveals that the CCs ST-8 and ST-11, CCs ST-18, ST-19 and ST-364, as well as the CCs ST-32, ST-35 and ST-269, respectively, are highly related. With the exception of GG-VI comprising exclusively ST-41/44 CC strains, almost all groups defined via mCGH are in turn lost when clustered according to conventional MLST (Figure 3 B). The latter holds true whether the strains are grouped using a majority rule consensus tree (Figure S1), or using an approach based on the concatenation of MLST gene sequences (Figure 3 B). The meningococcal population therefore displays a level of genetic structuring that cannot be detected using conventional MLST. In line with previous findings by others and with the estimated ρ/θ>1, the numerous cycles in the MLSA-based neighbor-net graph again indicate frequent recombination resulting in allelic conversion in housekeeping genes which might therefore be more frequent than the lateral transfer of entire genes leading to changes in gene content as detected by mCGH.

### Novel types of serogroup switching as suggested by mCGH

Despite ongoing recombination, there is an astonishing linkage between capsular serogroups and clonal complexes [7]. Capsule switching nevertheless has been reported and receives special attention because of its impact on vaccination strategies. Microevolution resulted in serogroup B to C [28], [29] and serogroup C to W-135 switches [30], [31]. Accordingly, strain α4 might represent a recent switch event from Y to W-135, as it is highly related to the ST-23 CC, which predominantly is associated with serogroup Y strains (Figure 3 A). This switch theoretically is easily accomplished by a single amino acid change of the capsule polymerase gene at amino acid position 310 [32], [33]. Another recent switch might explain the position of serogroup B strain DE8638 which clusters close to the two serogroup A strains and is clearly separated from all other serogroup B/C strains. This is most parsimoniously explained either by LGT of the entire *cps* region A encoding the serogroup B capsule synthesis genes from a serogroup B donor to a serogroup A recipient strain, resulting in a serogroup A to serogroup B capsule switch. More interestingly, the analysis of the population structure based on gene content further suggests common ancestors of serogroup 29E and W-135 meningococci which possibly diverged in earlier times and are now relatively stably associated to ST-60 CC and ST-22 CC (Figure 2 and 3 A). Strain α275 as a typical representative of W-135 strains from healthy carriers (ST-22 CC) [1] takes an intermediate position between serogroup Y and 29E and reflects a common descent of lineages expressing these serogroups. In all events, LGT of region A genes seems to have played a decisive role, and it was accordingly shown that this region is indeed part of a MME [26].

### Association between meningococcal gene content and pathogenicity

With respect to gene content differences, there was no significant association between the presence of certain virulence-associated genes and the source of the strain, i. e., whether it was isolated from a healthy carrier or from a case of invasive meningococcal disease. Although 64 of the 98 candidate virulence genes represented on the microarray were present in all meningococcal strains, some of the remaining 34 genes were differentially distributed between strains belonging to hyperinvasive lineages and carriage strains (see Figure S2). In particular, in line with previous findings [9] we found a significant association between genes located on Nf prophages and hyperinvasive lineages comprising, alongside Nf1/MDA genes coding mostly for hypothetical proteins, also Nf2-located genes including the gene coding for a zonula occludens toxin (zot)-like protein (Table 2). Furthermore, there is also a significant association between the hyperinvasive lineages and the genomic island IHT-E which predominantly encodes for proteins with poorly defined functions. Finally, we also found a significant association between hyperinvasive lineages and the presence of genes located on IHT-B including two genes that code for alternative TpsA C-terminal cassettes. TPS systems are composed of a secreted TpsA proteins and its cognate transporter TpsB [34], and TpsA proteins are translocated across the meningococcal outer membrane by their cognate transporters TpsB. Since it was recently shown that TpsA contributes to the interaction of meningococci with epithelial cells, differences in the repertoire of TpsA proteins are expected to result in differences in the interaction with host cells [35]. The eight GG-II and GG-III strains which comprise only carriage strains all lack a Nf1-encoded TspB gene and most lack also all three TpsA genes, TonB-dependent receptor genes as well as genes for FrpA/C activating enzymes. In contrast to these potential virulence-associated genes, the two “clustered regularly interspaced short palindromic repeats” (CRISPR)-associated genes *cas1* and *cas2* are significantly associated with carriage strains in the sample population. In many bacteria and archaea, these hypervariable loci take up genetic material from invasive elements and build up inheritable DNA-encoded immunity over time by targeting virus or plasmid nucleic acid in a sequence-specific manner [36].

**Table 2:** Genes significantly associated with hyperinvasive lineages.

Gene	Function(1)	OR(2)	95%-CI(3)	p(4)
**IHT-B**				
NMB0369	Conserved protein with hedgehog/intein (Hint) domain	n. d.(5)	3.82 - ∞	0.008
NMB0502	TpsA C-terminal cassette TpsS3	n. d.	3.82 - ∞	0.009
NMB0506	TpsA C-terminal cassette TpsS4	14.33	1.96 - 189.05	0.041
NMB0508	Conserved hypothetical protein	14.33	1.96 - 189.05	0.042
**Phage λ/IHT-E**				
NMB0899	Phage associated conserved protein	20.32	2.62 - 287.23	0.014
NMB0900	Putative KilA-N domain-containing protein	31.6	3.64 - 523.63	0.009
NMB0901	Conserved hypothetical protein	20.32	2.62 - 287.23	0.014
NMB0903	Conserved hypothetical cytoplasmic protein	20.32	2.62 - 287.23	0.015
NMB0904	Hypothetical periplasmic protein	20.32	2.62 - 287.23	0.016
NMB0906	Phage associated conserved protein	20.32	2.62 - 287.23	0.017
NMB0907	Conserved hypothetical protein	20.32	2.62 - 287.23	0.018
NMB0910	Putative phage HTH-type transcriptional regulator	20.32	2.62 - 287.23	0.019
NMB0916	Putative membrane protein (fragment)	20.32	2.62 - 287.23	0.020
**Nf1 phages**				
NMB1543	Putative phage replication initiation factor	20.32	2.62 - 287.23	0.021
NMB1544	Conserved hypothetical protein	41.48	3.87 - 2316.04	0.009
NMB1545	Hypothetical cytoplasmic protein	41.48	3.87 - 2316.04	0.011
NMB1546	Hypothetical integral membrane protein	41.48	3.87 - 2316.04	0.013
NMB1547	Hypothetical integral membrane protein	41.48	3.87 - 2316.04	0.018
NMB1550	Hypothetical integral membrane protein	29.64	2.88 - 1603.74	0.018
NMB1630	Hypothetical integral membrane protein	41.48	3.87 - 2316.04	0.026
**Nf2 phages**				
NMB1749	Putative zonular occludens toxin-like protein	n. d.	3.10 - ∞	0.013
NMB1750	Putative pilin gene-inverting protein (PIVML)	n. d.	4.00 - ∞	0.010
**CRISPR locus**				
NMO0346	CRISPR-associated protein Cas2	0.07	0.01 - 0.50	0.044
NMO0347	CRISPR-associated protein Cas1	0.05	0.00 - 0.38	0.023

(1) The functional annotation was taken from the NeMeSys database [69].

(2) Odds ratio of a Fisher's exact test.

(3) 95%-confidence interval of the respective odds ratio.

(4) P-values were computed upon the simultaneous comparison of 1679 genes using Fisher's exact test with the Benjamini-Hochberg multiple testing correction.

(5) n. d., not defined.

Comparative genome hybridization also allowed the identification of genes that are shared only among the pathogenic representatives of a particular GG. For example, GG-VII comprises five strains from hyperinvasive lineages as well as strain α522 from the non-hyperinvasive lineage ST-35 which is a three locus variant of ST-32. Compared to the other strains in this group, α522 lacks at least 25 genes that are primarily located on IHT-B and –C, suggesting that this strain might have lost substantial parts of these IHTs (Figure 2 and Table S4). In particular, α522 lacks three genes that encode hemagglutinin/hemolysin-related proteins which belong to TPS systems (TpsA1/NMB0493, TpsA2/NMB1768 and TpsB2/NMB1762) [37], thus emphasizing again the importance of TPS proteins for meningococcal virulence. In addition, strain α522 also lacks a FrpA/C-like protein on RTX island I [37] as well as genes located on the prophage Nf2-B3 [27] coding for the putative virulence factor TspB [9] and a zot-like protein. Also the content of the *pheS/pheT* locus differs between α522 and the other five strains in GG-VII, suggesting that the encoded type II restriction-modification system might limit gene exchange between α522 and the other strains and therefore contribute to the genetic differentiation observed in GG-VII.

### Recombination within the core genome

To assess the impact of homologous recombination on the meningococcal core genome we used the annotated (draft) genome sequences of seven strains from the sample population (Table 1). We analyzed a subset comprising 1092 of the 1139 core genes identified via mCGH that were also present and annotated in the seven (draft) genomes, and we found that 459 core genes (39.6%) have detectable signs of recombination using the pairwise homoplasy index Φ*_w_* [38] (Table S5). In line with their supporting role in DNA uptake and recombination in neisserial species, significantly more core genes with DUSs than core genes lacking DUSs showed signs of recombination (OR = 1.70, CI_0.95_ = [1.22, 2.37], p = 0.0011, Fisher's exact test), and compared to the accessory genome the meningococcal core genome is enriched for DUS containing genes (OR = 1.93, CI_0.95_ = [1.34, 2.81], p<0.001, Fisher's exact test). Remarkably, the distribution of core genes with and without signs of recombination among the different functional classes according to the COG classification scheme is significantly different (p<0.001, χ^2^ test). The recombining core genes are enriched for metabolic functions (p<0.01, Fisher's exact test) and accordingly are more often located in the cytoplasm (p<0.001, Fisher's exact test). Almost all metabolic pathways are affected by recombination, including the major pathways for energy conversion. These include enzymes involved in five of the seven steps required for the conversion of α-D-Glucose-6-phosphate (α-D-Glu-6-P) into phosphoenolpyruvate in gluconeogenesis/glycolysis, four of the ten enzymes of the citric acid cycle (TCA), all enzymes involved in the conversion of α-D-Glu-6-P into D-Glycerinaldehyde-3-phosphate via the pentose phosphate pathway, and seven of the 14 subunits of the NADH dehydrogenase. Likewise, also numerous genes involved in replication, recombination and repair of DNA showed signs for recombination. In particular, recombination in genes which are involved in homologous recombination such as *recA* and *recD* might feedback on the ability of the respective strain to properly incorporate foreign DNA which might in turn also affect the strains' genetic stability and evolvability. Table 3 gives further examples of putatively recombining core genes that are involved in basic biological processes.

**Table 3:** Selected examples of recombinant housekeeping genes from the core genome.

Gene(1)	Function(2)	Pathway/Category
**(Trace) element acquisition and coenzyme metabolism**		
*bioA*	Adenosylmethionine-8-amino-7-oxononanoate aminotransferase	Biotin
*bioD*	Aethiobiotin	Biotin
*folC*	Bifunctional tetrahydrofolate and dihydrofolate synthase	Folic acid
*hemA*	Glutamyl-tRNA reductase	Heme, porphyrin
*hemB*	Delta-aminolevulinic acid dehydratase	Heme, porphyrin
*fetB*	Enterobactin uptake system binding lipoprotein FetB	Iron acquisition
*fetE*	Putative ferric enterobactin uptake system ATP-binding protein FetE	Iron acquisition
*nicB*	Quinolinate synthetase B protein	Nicotinamide adenine dinucleotide
*pdxJ*	Pyridoxal phosphate biosynthetic protein PdxJ	Pyridoxine
*ribD*	Riboflavin biosynthesis protein RibD	Riboflavin, FAD/FMN
*ribF*	Riboflavin biosynthesis protein	Riboflavin, FAD/FMN
*metK*	S-adenosylmethionine synthetase	S-Adenosyl methionine biosynthesis
*cysA*	Sulfate/thiosulfate import ATP-binding protein CysA	Sulphur acquisition
*cysT*	Sulfate transport system permease protein CysT	Sulphur acquisition
*cysW*	Sulfate transport system permease protein CysW	Sulphur acquisition
**Conversion of biological energy**		
*eno*	Enolase (2-phosphoglycerate dehydratase)	Glycolysis
*fba*	Fructose-bisphosphate aldolase	Glycolysis
*fbp*	Fructose-1,6-bisphosphatase	Glycolysis
*gapA*	Glyceraldehyde 3-phosphate dehydrogenase A	Glycolysis
*pgi-1/-2*	Glucose-6-phosphate isomerase 1/2	Glycolysis
*pgk*	Phosphoglycerate kinase	Glycolysis
*pgm*	Phosphoglucomutase	Glycolysis
*pykA*	Pyruvate kinase II	Glycolysis
*nuoC*	NADH-quinone oxidoreductase chain C	Oxidative phosphorylation
*nuoD*	NADH-quinone oxidoreductase chain D	Oxidative phosphorylation
*nuoE*	NADH-quinone oxidoreductase chain E	Oxidative phosphorylation
*nuoF*	NADH-quinone oxidoreductase chain F	Oxidative phosphorylation
*nuoH*	NADH-quinone oxidoreductase chain H	Oxidative phosphorylation
*nuoJ*	NADH-quinone oxidoreductase chain J	Oxidative phosphorylation
*nuoN*	NADH-quinone oxidoreductase chain N	Oxidative phosphorylation
*tktA*	Transketolase (TK)	Pentose phosphate pathway
*fumA*	Fumarate hydratase class I	TCA cycle
*fumC*	Fumarate hydratase class II	TCA cycle
*lpdA*	Dihydrolipoyl dehydrogenase	TCA cycle
**Maintenance and replication of biological information**		
*mutY*	A/G-specific adenine glycosylase	Base excision repair
*tag*	DNA-3-methyladenine glycosylase I	Base excision repair
*ung*	Uracil-DNA glycosylase	Base excision repair
*dnaB*	Replicative DNA helicase	DNA replication
*rnhB*	Ribonuclease HII (RNase HII)	DNA replication
*ligA-1*	DNA ligase	DNA replication and repair
*dnaQ*	DNA polymerase III epsilon subunit	DNA replication, mismatch repair
*dnaZX*	DNA polymerase III tau/gamma subunits	DNA replication, mismatch repair
*recA*	RecA protein (recombinase A)	Homologous recombination
*recD*	Exodeoxyribonuclease V alpha chain	Homologous recombination
*recG*	ATP-dependent DNA helicase RecG	Homologous recombination
*ruvA*	Holliday junction DNA helicase RuvA	Homologous recombination
*ruvB*	Holliday junction DNA helicase RuvB	Homologous recombination
*ruvC*	Crossover junction endodeoxyribonuclease RuvC	Homologous recombination
*uvrD*	DNA helicase II	Mismatch and base excision repair
*xseB*	Exodeoxyribonuclease VII small subunit	Mismatch repair

(1) Gene abbreviations are based on the respective KEGG entries.

(2) The functional annotation was taken from the NeMeSys database [69].

## Discussion

To investigate the genomic basis of virulence, mCGH analyses of isolates from patients and healthy carriers are particularly useful for pathogens for which a suitable animal model of disease is lacking such as for *N. meningitidis* [39]. Consequently, meningococcal mCGH studies have already provided valuable insights into the genetic basis of virulence in this accidental pathogen [9], [16], [17], [18]. However, all these studies have some shortcomings with respect to the set of genes represented on the microarray, the breadth of the population genetic data provided along with the mCGH results, or the size and composition of the sample population. Here, we used a temporally and spatially well defined sample of the meningococcal population and provide MLST data for all strains analyzed to allow for a thorough comparison with the mCGH results. In addition, the microarray used to generate the mCGH data included genes from encapsulated serogroup A, B and C strains as well from the un-encapsulated strain α14. To systematically estimate the impact of recombination on the composition of the meningococcal core as well as accessory genomes, we further combined mCGH analyses with sequence-based computational approaches.

### Mobile genetic elements and the evolution of the meningococcal gene-pool

Our data indicate that MGEs and in particular bacteriophages have a major impact on population structure and virulence evolution in *N. meningitidis* thus confirming and extending previous findings by Hotopp *et al.* (2006) [16] and Bille *et al.* (2005) [9]. This is supported by the finding (i) that the presence of a λ-like prophage or the derived genomic island IHT-E [16] splits the sample population into two major groups, one comprising GG-I and GG-V–GG-VIII and the other comprising GG-II–GG-IV (Figure 2 and 3A), and (ii) that certain genes located at CRISPR loci are more often found in non-hyperinvasive lineages whereas (iii) the Nf prophages are more often found in hyperinvasive lineages (Table 2). However, the possible contribution of the integrated prophages to the fitness of the transduced strain is poorly understood and might in fact be different for different prophages. For example, it has recently been shown by mathematical modeling that horizontally acquired genes can persist for a long time in a substantial fraction of individuals in the population even when they are neutral or slightly deleterious [40]. Therefore many prophages found in the meningococcal genomes such as the abundant Mu-like prophages might constitute merely parasitic DNA. Alternatively, some prophages such as the Nf1/MDA prophage might confer a fitness advantage as they might act as mutators on meningococcal chromosome structure [14], [27] and thereby increase the genetic variability of the meningococcal population. This increase in genetic variability can be selected for during adaptation via second-order selection [41] and can result in an improved adaptability of the transduced bacteria [42]. However, the possible contribution of (pro)phages to meningococcal fitness and virulence awaits further experimental investigation.

In addition to the integration of prophages into the genomes, lateral transfer of MMEs also contributes substantially to meningococcal gene-complement diversity, and about one-third of the strain-specific genes and half of the genes that are specifically present in only one of the eight GGs are located on (c)MMEs (Table S2). Their high mobility is witnessed by the fact that even strains from the same ST differ in their complement of (c)MMEs (Table S3). Since it has recently been suggested that MMEs are involved in LGT in *Neisseria* and in other bacterial species [26], we hypothesize that lateral transfer of MMEs genes from bacterial species residing in the human nasopharynx might contribute to gene complement differences among different meningococcal strains. Whole-genome sequences from larger meningococcal strain collections in conjunction with ongoing metagenomic efforts will allow assessing the extent of LGT between meningococci and other species of the human nasopharyngeal mircobiome [43].

### Meningococcal population structure as revealed by mCGH studies

Given the differences in the microarray platforms used, the differences in the breadth of strains examined and dissimilar analysis methods, it is quite remarkable that grouping of strains based on gene content in this study resulted in clusters similar to the ones found by Hotopp *et al.* (2006) [16] which is so far the most comprehensive study with respect to the number of strains investigated via mCGH (n = 48). In particular, comparison of the mCGH groupings of the three strains FAM18, MC58, and Z2491 that were included in both studies allowed to correlate mCGH group mCGH-2 defined by Hotopp *et al.* (2006) with GG-IV, mCGH-3 with GG-VIII and mCGH-5 with GG-VII, respectively. Similar to GG-IV, mCGH-2 comprises almost exclusively serogroup A strains whereas mCGH-3 as well as GG-VIII comprise exclusively serogroup C strains and mCGH-5 like GG-VII comprehends only serogroup B strains, respectively. Based on the mCGH groups for those 22 strains for which Hotopp *et al.* (2006) provide MLST data, both studies also demonstrate a similar clustering of CCs into groups based on gene content. For example, almost all ST-41/-44 CC strains cluster together and likewise all ST-8 and ST-11 CC strains. Given the high genome variability of meningococci as well as the differences with respect to time of isolation and geographical spread, both strain collections therefore show a remarkably similar population structure. This suggests (i) that meningococcal population structure can be reproducibly analyzed via mCGH, and (ii) that genetic structuring might be quite stable over time and highly similar for samples taken from different geographic regions.

### Recombination and virulence in *N. meningitidis*

Evolutionary pressures exerted by the host on bacterial proteins important for virulence are often computationally quantified by the ratio of substitution at non-synonymous (dN) and synonymous sites (dS) (e. g. [44]). However, it was recently shown that dN/dS is time dependent for closely related bacterial genomes [45] and that it might even be impossible to infer selection pressures from such data for population samples from the same species [46]. Therefore, to identify proteins involved in meningococcal pathogenesis, the analysis of recombination signals in protein coding genes might provide an alternative approach since genomic regions coding for proteins with a role in pathogenicity were recently suggested to exhibit high rates of recombination [47]. As demonstrated in the preceding sections, recombination has indeed a pervasive effect on the meningococcal core genome with about 40% of the genes investigated showing signs of (intragenic) recombination. The finding that recombinant genes are enriched for genes coding for metabolic functions would further imply that in *N. meningitidis* differences in metabolism might contribute to virulence. This hypothesis is supported by recent epidemiological findings showing that via small differences in metabolic efficiency, co-adapted combinations of housekeeping gene alleles are associated with differences in meningococcal transmission fitness [48]. Such small differences in transmission fitness were suggested to increase the diseases incidence caused by the respective strains [49], thus imparting a central role for housekeeping genes in meningococcal virulence. In line with this hypothesis, we found that, e.g., seven of the 14 genes encoding the subunits of the meningococcal NADH dehydrogenase (quinone) complex have signs for intragenic recombination (Table 3). Since this multi protein complex catalyses a key step in oxidative phosphorylation, it is conceivable that sequence differences in this multi protein complex might affect the in vivo fitness and therefore virulence differences among different CCs.

Our computational approach, however, provides only a lower estimate for the genome-wide impact of homologous recombination on the meningococcal core genome due to a number of reasons: (i) only seven meningococcal genomes were compared and it is likely that with increasing numbers of sequenced genomes also the percentage of genes positively tested for recombination will increase [38]; (ii) as the power to detect recombination increases as a function of sequence length [50] pre-processing of alignments as performed in this study further decreases the sensitivity to detect intragenic recombination; (iii) for large data sets multiple testing corrections further erode statistical power to the point that even relatively obvious recombination signals might be missed [51]; and finally (iv) recombination is not detectable in genes under strong purifying selection which purges any sequence variation required for the detection of intragenic recombination by most algorithms.

In line with previous findings by others [34], [52], our data suggest that, in addition to recombination-mediated differences in a large number of housekeeping loci within the core genome, genetic differences among hyperinvasive and non-hyperinvasive CCs likely comprise also different repertoires and allelic profiles of RTX toxin- and TPS protein-encoding genes (Table 2 and Table S4). Together with differences among strains in the repertoire of surface adhesins [13] and in the presence of chromosomally integrated Nf1/MDA prophages [9], these data re-emphasize and support the concept of a polygenic nature of meningococcal virulence.

## Materials and Methods

### Bacterial strains and sample preparation

The 16 carriage strains analyzed in this study were taken from the Bavarian carriage strain collection described in [1] whereas the ten contemporary disease isolates were from the strain collection of the NRZM (Würzburg, Germany) (Table 1). Strain selection was guided by the following criteria: (i) the selected strains should cover the most prevalent CCs found among carriers and disease cases as identified by MLST; (ii) wherever possible the founder ST was chosen for each CC according to the Neisseria MLST database (http://pubmlst.org/neisseria/) [53]; (iii) based on the epidemiological data for the CCs a carriage or disease strain or both were chosen to represent the respective complex; (iv) for each of the six serogroups represented in the data set at least two isolates from different CCs were chosen including also two capsule null locus (*cnl*) strains; and (v) strains MC58, Z2491 and FAM18 for which whole genome sequences were available were also included to complement the data from the mCGH experiments with data from computational genome comparisons.

### Computational analyses of MLST data

Alleles of the seven housekeeping genes *abcZ*, *adk*, *aroE*, *fumC*, *gdh*, *pdhC* and *pgm*, sequence types, and clonal complexes were assigned on the basis of the *Neisseria* MLST database. ClonalFrame version 1.1 [54] was used for grouping strains based on the sequences of individual genes. From the results of 10 independent runs with 100.000 iterations each and a burn-in period of 100.000 a 50% majority rule consensus tree was computed and visualized as a dot graph. Based on the alignments of the concatenated MLST gene fragments comprising 3284 sites with 383 parsimony informative sites SplitsTree4 [55] was used to construct a neighbor-net [56]. With jModelTest [57] the GTR+I+Γ model of nucleotide substitution was inferred from this data set using the “Bayesian information criterion” (BIC) with 6 rate categories, the proportion of invariable sites p-inv = 0.75 and the shape parameter of the Γ distribution α = 0.52 to model rate heterogeneity among sites. To assess the presence of recombination in the individual MLST loci we used the pairwise homoplasy index Φ*_w_* as implemented in the software PhiPack [38] as it was shown to reliably detect recombination even in the presence of substitution rate heterogeneity. DnaSP version 4 [58] was used to calculate Tajima's D [59], the pairwise nucleotide diversities (π) as well as the gene wise population recombination (ρ) [60] and mutation (θ) rates, respectively, and to detect genetic differentiation of subpopulations by calculating K_s_* and Z* [61] as well as S_nn_ [62] statistics with 1000 replicates to assess statistical significance in the permutation test. The latter three statistics were shown to be most powerful for small sample sizes and in the presence of recombination [61], [62]. RDP3 [63] was used for confirmation of recombination signals within MLST gene alignments.

### Microarray hybridization and data processing

For mCGH analyses, chromosomal DNA was isolated from bacteria grown in 5 ml of Proteose Peptone Medium supplemented with 1% Polyvitex (Biomereux) (PPM+) using QIAGEN Genomic-tip 20/G (Qiagen, Hilden, Germany). Aliquots of all genomic DNAs to be tested were pooled to form the common reference, 4 µg of test DNA was labeled with Cy3 dCTP (GE healthcare, Munich, Germany) and 4 µg of reference DNA was labeled with Cy5 dCTP (GE healthcare, Munich, Germany) using Klenow Enzyme as described in [20]. The 70mer oligonucleotide-based microarrays representing the genomes of the meningococcal strains α14 [14], FAM18 [12], MC58 [64] and Z2491 [65], respectively, were pre-hybridized according to the manufacturer's protocols (Schott AG, Germany). Labeled DNAs were hybridized onto the microarray slides using a Tecan HS 4800™ Pro hybridization station (Tecan Deutschland GmbH, Crailsheim, Germany). Three microarrays were performed for each probe. The slides were scanned using Genepix 4200 and the raw data files were extracted using Genepix Pro 4.0. Spots were flagged in obvious instances of high background or stray fluorescent signals. Hybridization data were further processed using VSN normalization and Limma [66] implemented in the R language [67]. The normalized intensities were used for absence/presence prediction of individual genes as described in [20]. Since the microarray was originally designed for transcriptome analyses post-processing of mCGH hits was carried out as described in Schwarz et el. (2010) [20] to further improve the signal-to-noise ratio. This allowed for the simultaneous assessment of the presence of 1679 genes with an overall accuracy of 98%, a type I error rate of 5% and a type II error rate of 1% [20]. All comparative genome hybridization data is MIAME compliant and the raw data has been deposited in the Gene Expression Omnibus (GEO) database under accession number GSE18078.

### Computational analyses of mCGH data

The functional classification of core, distributed and strain-specific genes as identified by mCGH was based on the COG classification scheme [68]. CAI values, GC content and the assignments of the subcellular localization of encoded proteins were taken from the NeMeSys database [69]. MMEs and candidate (c)MMEs were identified based on the criteria given in [26], [70], and the phage designations for strains α14, FAM18, MC58 and Z2491 were taken from [12], [14], [27], [64], [65], [71]. Genomic regions that showed an atypical low GC content and that did not display the hallmark features of MMEs, canonical genomic islands [72] or bacteriophages were classified as islands of horizontal transfer according to [64].

For strain clustering based on gene content, maximum parsimony was used as implemented in Paup* 4.0 [73] with the heuristic tree search option and the DELTRAN option for character state optimization. For the generation of a phylogenetic network using the neighbour-net algorithm [56], pairwise genome distances were computed based on gene content using SplitsTree4 [55]. To assess the statistical robustness of the phylogenetic reconstructions, bootstrap analyses were performed with 1000 resampling steps.

For each gene, Fisher's exact test with the Benjamini-Hochberg (BH) multiple testing correction [74] was used to test for a possible association with hyperinvsive and carriage lineages, respectively.

### Genome-wide screen for intragenic recombination in core genes

Annotated .gbk files of *N. meningitidis* strains FAM18 (AM421808) [12], MC58 (AE002098) [64] and Z2491 (AL157959) [65] (Table 1) were downloaded via ftp from the National Center for Biotechnology Information database (http://www.ncbi.nlm.nih.gov/genomes/lproks.cgi). The annotated genome sequences of *N. meningitidis* strains α14 (AM889136) [14], α153 (AM889137) [14], α275 (AM889138) [14] and α710 (CP001561) were taken from the in-house GenDB [75] database. Orthologous proteins were operationally identified as bidirectional best hits [76] with more than 50% amino acid sequence identity over at least 50% of the query sequence length, and for all groups of orthologous proteins we consecutively used a combination of MUSCLE [77], RevTRans [78] and Gblocks [79] to obtain gap-free codon-based nucleotide sequence alignments of the respective genes. The genomes of strains α153 and α275 were sequenced to on average 8× coverage by Sanger sequencing as described in [14], and the resulting 87 and 133 non-overlapping contigs, respectively, were pasted together into a pseudochromosome in random order with the sequence 5′-CTAGCTAGCTAG-3′ used as spacer that generates a stop codon in all six reading frames. Therefore, the draft-genome sequences of these two strains do not cover the entire chromosomes, and the computational analyses could thus only be performed on a subset of 1092 of the 1139 genes (95.9%) that were found by mCGH to be present on all 29 strains. Since BIC based model inference further indicated mutation rate heterogeneity in the concatenated MLST gene data set comprising the entire set of 29 strains as outlined above, we calculated Φ*_w_* using PhiPack [38] to screen for signs of intragenic recombination in the 1092 processed sequence alignments. Correction for multiple testing was performed using the procedure reported by Benjamini and Hochberg [74] to control the false discovery rate (FDR), and FDR values below 0.05 were used as indicative for the presence of recombination. For a sample of 50 genes given in Table 3 the presence of recombination in the respective alignments was also manually confirmed using RDP3 [63].

## Supporting Information

Figure S1 Genetic structure of the sample population based on MLST. Dot graph representation based on a majority rule consensus tree of the seven housekeeping gene fragments form the meningococcal core genome used for MLST calculated with ClonalFrame. Based on the sequence in housekeeping genes genomic groups as defined by mCGH are torn apart such as GG-II (dark green), GG-III (light blue) or GG-V (mauve). Likewise, also strains from the same serogroup such as 29E, W-135 or Y do not cluster.
(TIF)

Figure S2 Distribution of surface and virulence-associated proteins. Only surface and virulence-associated proteins are shown that are variably present among the 29 meningococcal strains compared. The respective genes were taken from recent compilations given in refs. [14], [84].
(TIF)

Table S1 Overview of the important population genetic data of the sample population.
(DOC)

Table S2 Genes specifically present or absent in only one genome group.
(DOC)

Table S3 Genes differently distributed between pairs of strains from the same ST.
(DOC)

Table S4 Genes specific for strains from hyperinvasive lineages within GG-VI.
(DOC)

Table S5 Core genes of the sample population with significant evidence for recombination in the Φ_W_ statistic.
(DOC)
