# Supplementary figures and images for: Virulence Evolution of the Human Pathogen Neisseria meningitidis by Recombination in the Core and Accessory Genome
Source: PLoS One. 2011 Apr 26;6(4):e18441. doi: 10.1371/journal.pone.0018441 (PMC3082526; doi:10.1371/journal.pone.0018441)

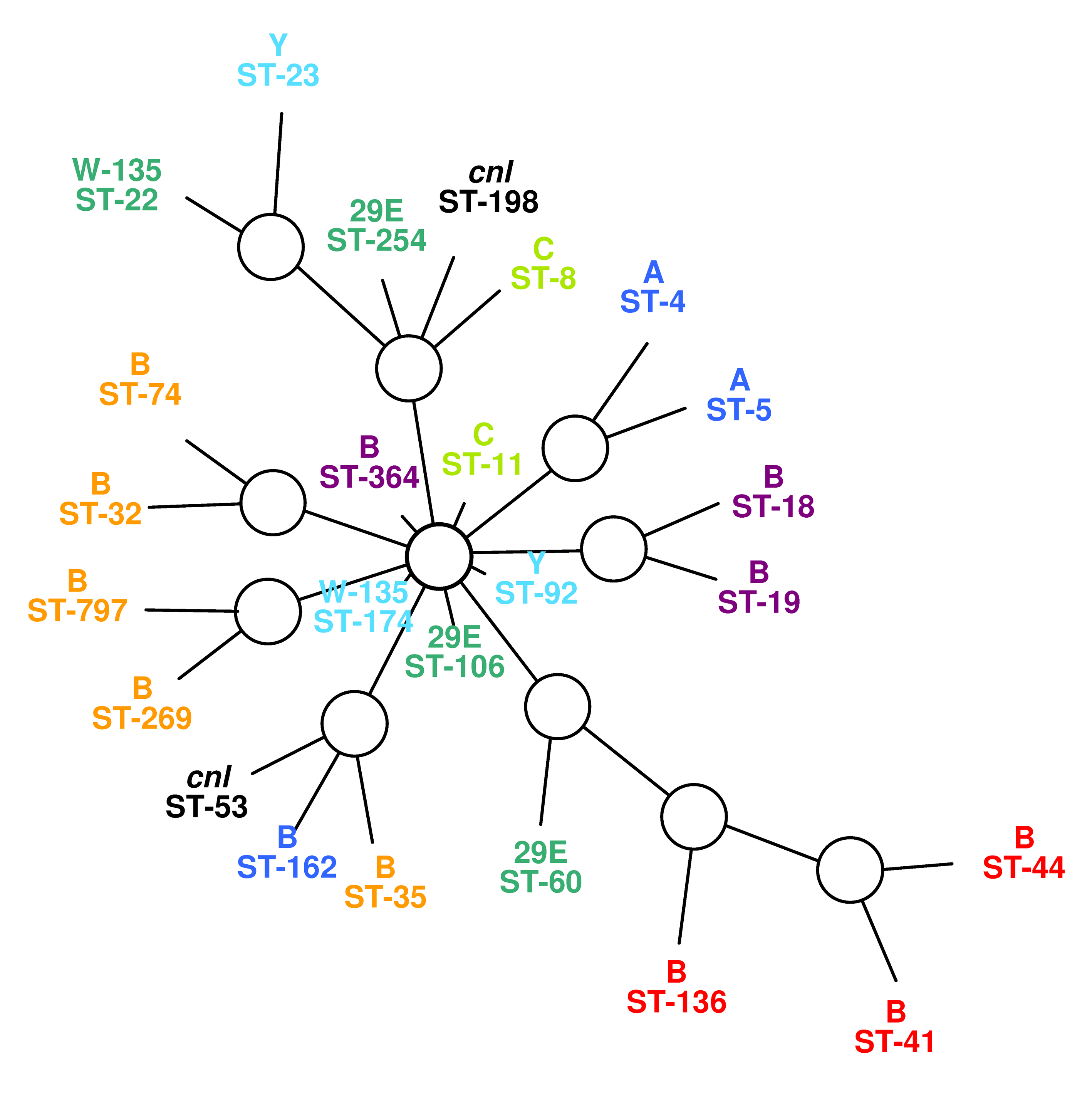

Supplement: Figure S1 — Genetic structure of the sample population based on MLST. Dot graph representation based on a majority rule consensus tree of the seven housekeeping gene fragments form the meningococcal core genome used for MLST calculated with ClonalFrame. Based on the sequence in housekeeping genes genomic groups as defined by mCGH are torn apart such as GG-II (dark green), GG-III (light blue) or GG-V (mauve). Likewise, also strains from the same serogroup such as 29E, W-135 or Y do not cluster. (TIF) [file pone.0018441.s001.tif]

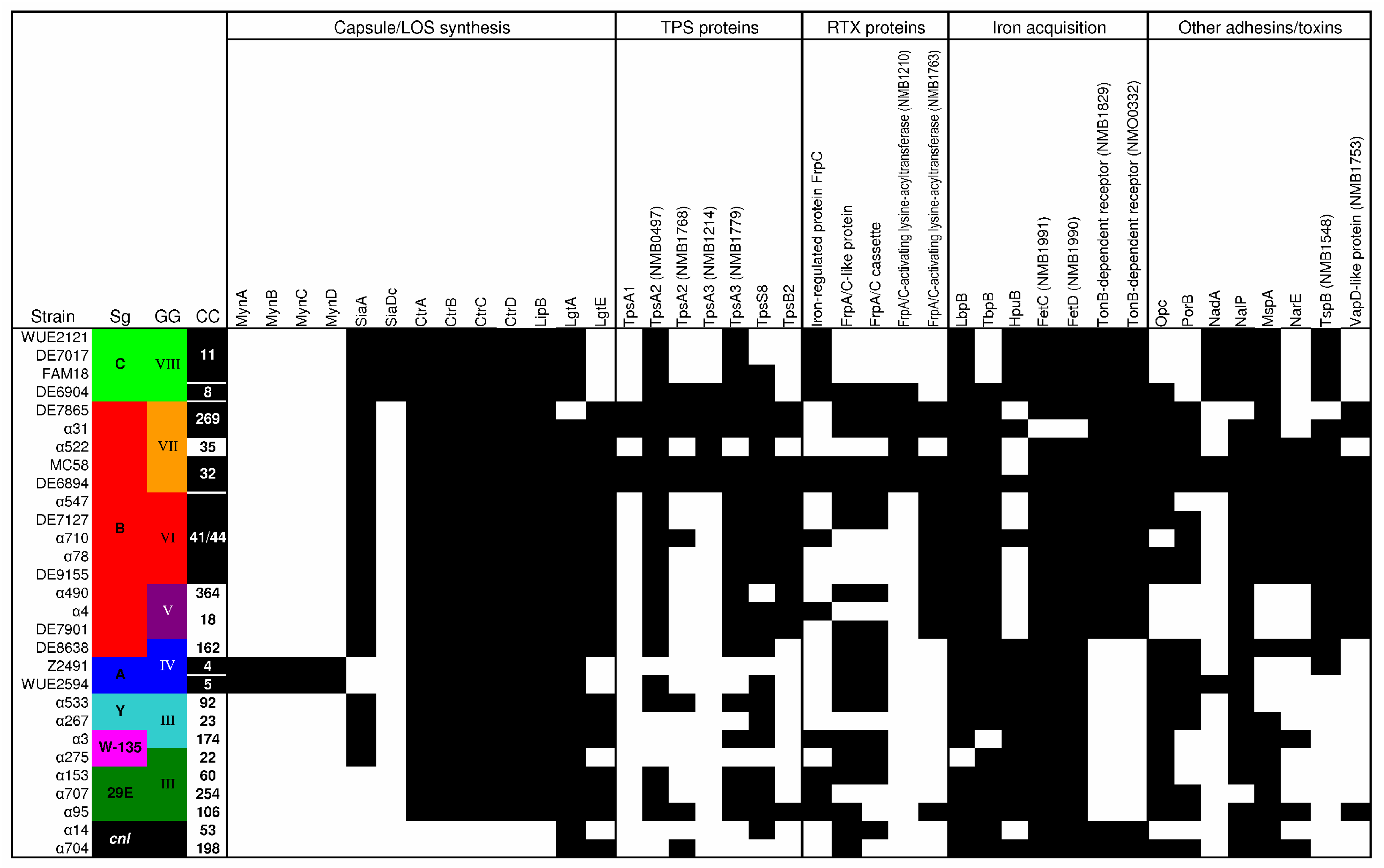

Supplement: Figure S2 — Distribution of surface and virulence-associated proteins. Only surface and virulence-associated proteins are shown that are variably present among the 29 meningococcal strains compared. The respective genes were taken from recent compilations given in refs. [14], [84]. (TIF) [file pone.0018441.s002.tif]
