# Supplementary material for: Virulence Evolution of the Human Pathogen Neisseria meningitidis by Recombination in the Core and Accessory Genome
Source: PLoS One. 2011 Apr 26;6(4):e18441. doi: 10.1371/journal.pone.0018441 (PMC3082526; doi:10.1371/journal.pone.0018441)
Supplement: Table S1 — Overview of the important population genetic data of the sample population. (DOC) [file pone.0018441.s003.doc]

**Table S1**: Overview of some population genetic data of the sample population.

|  | *abcZ* | *adk* | *aroE* | *fumC* | *gdh* | *pdhC* | *pgm* |
| --- | --- | --- | --- | --- | --- | --- | --- |
| Number of sites | 433 | 465 | 490 | 465 | 501 | 480 | 450 |
| Number of variable sites (S) | 80 | 17 | 166 | 37 | 23 | 76 | 67 |
| Number of haplotypes (h) | 15 | 10 | 17 | 17 | 11 | 17 | 13 |
| Average number of nucleotide differences (k) | 21.6 | 4.6 | 43.1 | 9.9 | 8.0 | 18.1 | 19.8 |
| Estimate of mutation rate per gene (θ) | 21.6 | 4.6 | 43.1 | 9.9 | 8.0 | 18.1 | 19.8 |
| Estimate of recombination rate per gene (ρ) | 18.0 | 13.2 | 3.8 | 12.2 | 22.2 | 4.7 | 13.3 |
