# Supplementary material for: Virulence Evolution of the Human Pathogen Neisseria meningitidis by Recombination in the Core and Accessory Genome
Source: PLoS One. 2011 Apr 26;6(4):e18441. doi: 10.1371/journal.pone.0018441 (PMC3082526; doi:10.1371/journal.pone.0018441)
Supplement: Table S2 — Genes specifically present or absent in only one genome group. (DOC) [file pone.0018441.s004.doc]

**Table S2**: Genes specifically present (+) or absent (-) in only one genome group.

| **Gene** | **Location** | **Presence** | **Function^(1)^** |
| --- | --- | --- | --- |
| **GG-I** |  |  |  |
| NMB0071 | IHT-A/*cps* | - | Polysialic acid capsule export outer-membrane lipoprotein CtrA |
| NMB0072 | IHT-A/*cps* | - | Polysialic acid capsule export inner-membrane protein CtrB |
| NMB0077 | IHT-A/*cps* | - | Truncated adenine-specific methyltransferase |
| NMB0082 | IHT-A/*cps* | - | Polysialic acid capsule modification protein LipA |
| NMB0083 | IHT-A/*cps* | - | Polysialic acid capsule modification protein LipB |
| NMB0459 | NIME array^(2)^ | - | Filamentation induced by cAMP (Fic)-family protein |
| **GG-II** |  |  |  |
| NMB1623 | cMME*norB*^(3)^ | - | Copper-type nitrite reductase AniA |
| NMB1624 | cMME*norB*^(3)^ | - | Sulphatase-modifying factor |
| **GG-V** |  |  |  |
| NMB1033 | (cMME*leuD*) | - | Type II restriction-modification methylase M.NlaIV |
| NMB1740 | Nf2-B3 | - | Hypothetical protein |
| **GG-VII** |  |  |  |
| NMB1329 | MME*trmBuvrB* | + | Conserved protein of unknown function |
| NMB1330 | MME*trmBuvrB* | + | Conserved hypothetical protein |
| NMB2008 | MMENMB2007-10 | + | ABC transporter ATP-binding protein |
| NMB2012 | MME*bfrAlipA* | + | HTH-type transcriptional regulator |
| NMB2013 | MME*bfrAlipA* | + | Conserved hypothetical protein |
| **GG-VIII** |  |  |  |
| NMC0050 | IHT-A/*cps* | + | Capsular polysaccharide O-acetyltransferase OatC |
| NMC0678 | MME*pheSpheT* | + | Very short patch repair endonuclease V.NmeDI |
| NMC0679 | MME*pheSpheT* | + | Cytosine-specific methyltransferase M.NmeDI |
| NMC0855 | Phage λ/MGI-1 | + | Conserved bacteriophage protein |
| NMC0939 | MME*sucDuvrA* | + | Conserved hypothetical protein |
| NMB1092 | Phage Pnm2 | - | DNA polymerase III beta subunit |

^(1)^The functional annotation was taken from the NeMeSys database [94].

^(2)^The gene is located in a NIME repeat array upstream of the *tbpBA* genes in MC58.

^(3)^The corresponding genes are located between *norB* and *opaB* and flanked by two NIME repeats in the genome of α14.
