# Supplementary material for: Virulence Evolution of the Human Pathogen Neisseria meningitidis by Recombination in the Core and Accessory Genome
Source: PLoS One. 2011 Apr 26;6(4):e18441. doi: 10.1371/journal.pone.0018441 (PMC3082526; doi:10.1371/journal.pone.0018441)
Supplement: Table S3 — Genes differently distributed between pairs of strains from the same ST. (DOC) [file pone.0018441.s005.doc]

**Table S3.** Genes differently distributed between pairs of strains from the same ST.

| **Gene** | **Location** | **Function^(1)^** | **Variation^(2)^ within** | | |
| --- | --- | --- | --- | --- | --- |
|  |  |  | **ST-11** | **ST-41** | **ST-44** |
| **MMEs** |  |  |  |  |  |
| NMA0408 | MME*mtr* | Conserved hypothetical membrane protein |  |  | x |
| NMA0640 | MME*pglC* | Putative pilin glycosyltransferase | x | x |  |
| NMB0119 | cMME*topA* | Hypothetical protein |  | x |  |
| NMB0835 | MME*rfaDclpA* | Truncated type I restriction-modification system R protein | x |  |  |
| NMB1264 | MME*zurcobW* | Conserved hypothetical protein (pseudogene) |  | x |  |
| NMB1265 | MME*zurcobW* | Conserved hypothetical protein |  | x |  |
| NMB1596 | MME*alaSgpm* | Conserved protein of unknown function |  | x |  |
| NMB1825 | MME*dnaE* | Hypothetical protein | x |  |  |
| NMB1826 | MME*dnaE* | Conserved protein with RNA-binding S4 domain | x |  |  |
| **Phages** |  |  |  |  |  |
| NMA1172 | Nf2-A1 | Conserved protein of unknown function |  |  | x |
| NMA1173 | Nf2-A1 | Truncated TspB-like protein (N-terminal third) |  |  | x |
| NMA1307 | Pnm2 | Hypothetical protein | x |  |  |
| NMB1110 | Pnm2 | Putative phage tail protein | x |  |  |
| NMB1543 | Nf1-B1 | Putative phage replication initiation factor | x |  |  |
| NMB1747 | Nf2-B3 | Putative TspB protein | x |  |  |
| NMC1713 | Nf1-C3 | Hypothetical integral membrane protein |  | x | x |
| NMC1715 | Nf1-C3 | Putative TspB protein |  | x | x |
| NMO0932 | NeisMu3 | Putative phage tail tape measure protein | x |  |  |
| **IHTs** |  |  |  |  |  |
| NMA1078 | IHT-F^(3)^ | Hypothetical protein | x |  |  |
| NMA1080 | IHT-F^(3)^ | Conserved protein of unknown function |  | x | x |
| NMB0855 | IHT-F^(3)^ | Putative peptidase |  | x | x |
| NMB0858 | IHT-F^(3)^ | Hypothetical protein |  |  | x |
| NMB0860 | IHT-F^(3)^ | Hypothetical protein |  |  | x |
| NMB1775 | IHT-C | Putative TpsS8 cassette | x |  |  |
| **Others** |  |  |  |  |  |
| NMA2121 | *maf1* locus | Hypothetical protein | x |  |  |
| NMC0605 | *maf3* locus | Conserved hypothetical protein | x |  |  |
| NMO0337 | CRISPR locus | Conserved hypothetical protein (pseudogene) | x |  |  |

^(1)^The functional annotation was taken from the NeMeSys database [94].

^(2)^Genes that are variably present in two strains from the same ST are denoted by an “x”.

^(3)^IHT-F is a region of unusually low GC content in strain Z2491 and comprises 16 genes (NMA1066 – NMA1084). While NMA1066 codes for a putative bactriocin processing peptidase (IPR005074) most other genes (10/16) code for hypothetical proteins, and no integrases or transposases are located on IHT-F.
