# Supplementary material for: Virulence Evolution of the Human Pathogen Neisseria meningitidis by Recombination in the Core and Accessory Genome
Source: PLoS One. 2011 Apr 26;6(4):e18441. doi: 10.1371/journal.pone.0018441 (PMC3082526; doi:10.1371/journal.pone.0018441)
Supplement: Table S4 — Genes specific for strains from hyperinvasive lineages within GG-VI. (DOC) [file pone.0018441.s006.doc]

**Table S4.** Genes specific for hyperinvasive lineages within GG-VII^(1)^.

| **Gene** | **Function^(2)^** |
| --- | --- |
| **IHT-B** |  |
| NMB0491 | Hypothetical protein |
| NMB0492 | Conserved hypothetical protein |
| NMB0493 | Haemagglutinin/hemolysin-related protein TpsA1 |
| NMB0495 | Putative replication initiation protein |
| NMB0503 | Hypothetical protein |
| **IHT-C** |  |
| NMB1753 | Putative VapD-like protein |
| NMB1754 | Cryptic plasmid protein A-related protein (artifact) |
| NMB1756 | Hypothetical protein |
| NMB1757 | Conserved protein of unknown function (artifact) |
| NMB1758 | Hypothetical protein |
| NMB1760 | Conserved hypothetical protein |
| NMB1761 | Conserved hypothetical protein |
| NMB1762 | TpsA2 activation/secretion protein TpsB2 |
| NMB1763 | Putative FrpA/C-activating lysine-acyltransferase |
| NMB1768 | Haemagglutinin/hemolysin-related protein TpsA2 |
| NMB1777 | Conserved hypothetical protein |
| **RTX island I** |  |
| NMB1403 | FrpA/C-like protein |
| NMB1405 | Putative FrpA/FrpC cassette |
| **Phage Nf2-B3** |  |
| NMB1747 | Putative TspB protein |
| NMB1749 | Putative zonula occludens toxin-like protein |
| NMB1750 | Putative pilin gene-inverting protein (PIVML) |
| **MME*pheSpheT*** |  |
| NMB0725 | Type II restriction-modification methylase M1-NmeBI |
| NMB0726 | Type II restriction-modification enzyme R-NmeBI |
| NMB0727 | Type II restriction-modification methylase M2-NmeBI |

^(1)^ No genes were found to be specifically present only in α522 with respect to the other strains in this group.

^(2)^ The functional annotation was taken from the NeMeSys database [94].
