# Supplementary material for: Virulence Evolution of the Human Pathogen Neisseria meningitidis by Recombination in the Core and Accessory Genome
Source: PLoS One. 2011 Apr 26;6(4):e18441. doi: 10.1371/journal.pone.0018441 (PMC3082526; doi:10.1371/journal.pone.0018441)
Supplement: Table S5 — Core genes of the sample population with significant evidence for recombination in the ΦW statistic. (DOC) [file pone.0018441.s007.doc]

**Table S5**: Core genes of the sample population with significant evidence for recombination in the Φ_W_ statistic.

| **Name** | ***Gene*** | **Product** |
| --- | --- | --- |
| NMA0189 | *rfbB1* | dTDP-glucose 4,6-dehydratase |
| NMB0002 |  | conserved hypothetical periplasmic protein |
| NMB0004 |  | conserved hypothetical periplasmic protein |
| NMB0006 |  | putative periplasmic thioredoxin |
| NMB0008 | *ftsX* | cell division protein FtsX |
| NMB0010 | *pgk* | phosphoglycerate kinase |
| NMB0011 | *murA* | UDP-N-acetylglucosamine 1-carboxyvinyltransferase (Enoylpyruvate transferase; UDP-N-acetylglucosamine enolpyruvyl transferase; EPT) |
| NMB0013 |  | conserved hypothetical integral membrane protein |
| NMB0014 | *kdtA* | 3-deoxy-D-manno-octulosonic-acid transferase (KDO transferase) |
| NMB0015 | *gnd* | 6-phosphogluconate dehydrogenase, decarboxylating |
| NMB0027 | *fkbP* | FK506-binding protein (peptidyl-prolyl cis-trans isomerase; PPIase; rotamase) |
| NMB0031 | *glmS* | glucosamine--fructose-6-phosphate aminotransferase [isomerizing] (hexosephosphate aminotransferase; D-fructose-6- phosphate amidotransferase; GFAT; L-glutamine-D-fructose-6-phosphate amidotransferase; glucosamine-6-phosphate synthase) |
| NMB0034 |  | conserved hypothetical integral membrane protein |
| NMB0036 |  | putative Tat-translocated enzyme |
| NMB0045 | *ftsY* | cell division protein FtsY |
| NMB0050 |  | conserved hypothetical integral membrane protein |
| NMB0053 |  | conserved hypothetical protein |
| NMB0062 | *rfbA* | glucose-1-phosphate thymidylyltransferase (dTDP-glucose synthase; dTDP-glucose pyrophosphorylase) |
| NMB0075 |  | putative RNA-binding protein |
| NMB0088 |  | conserved hypothetical protein |
| NMB0089 | *pykA* | pyruvate kinase II (PK-2) |
| NMB0106 | *pyrB* | aspartate carbamoyltransferase (aspartate transcarbamylase; ATCase) |
| NMB0111 | *fmt* | methionyl-tRNA formyltransferase |
| NMB0112 | *rmsB* | ribosomal RNA small subunit methyltransferase B (rRNA (cytosine-C(5)-)-methyltransferase; 16S rRNA m5C967 methyltransferase) |
| NMB0115 |  | putative two-component system transcriptional regulator protein |
| NMB0118 | *topA* | DNA topoisomerase I (Omega-protein; relaxing enzyme; untwisting enzyme; swivelase) |
| NMB0124 | *tuf* | elongation factor Tu (EF-Tu) |
| NMB0128 | *rplA* | 50S ribosomal protein L1 |
| NMB0145 | *rplB* | 50S ribosomal protein L2 |
| NMB0173 | *oxyR* | hydrogen peroxide-inducible genes transcriptional activator |
| NMB0176 | *dadA* | D-amino acid dehydrogenase small subunit |
| NMB0178 | *lpxA* | acyl-[acyl-carrier-protein]--UDP-N-acetylglucosamine O-acyltransferase (UDP-N-acetylglucosamine acyltransferase) |
| NMB0180 | *lpxD* | UDP-3-O-[3-hydroxymyristoyl] glucosamine N-acyltransferase |
| NMB0182 | *omp* | outer-membrane protein assembly factor Omp85 |
| NMB0183 |  | putative zinc metallopeptidase |
| NMB0184 | *dxr* | 1-deoxy-D-xylulose 5-phosphate reductoisomerase (DXP reductoisomerase; 1-deoxyxylulose-5-phosphate reductoisomerase) |
| NMB0186 | *uppS* | undecaprenyl pyrophosphate synthetase (UPP synthetase; di-trans,poly-cis-decaprenylcistransferase; undecaprenyl diphosphate synthase; UDS) |
| NMB0190 | *gidB* | methyltransferase GidB (glucose-inhibited division protein B) |
| NMB0192 | *rnhB* | ribonuclease HII (RNase HII) |
| NMB0198 | *rluC* | ribosomal large subunit pseudouridine synthase C (rRNA-uridine isomerase C; rRNA pseudouridylate synthase C) |
| NMB0203 | *dapB* | dihydrodipicolinate reductase (DHPR) |
| NMB0206 | *aat* | leucyl/phenylalanyl-tRNA--protein transferase (L/F- transferase; leucyltransferase; phenylalanyltransferase) |
| NMB0207 | *gapA* | glyceraldehyde 3-phosphate dehydrogenase A (GAPDH) |
| NMB0208 |  | putative ferredoxin |
| NMB0211 | *sda* | L-serine dehydratase (L-serine deaminase; SDH; L-SD) |
| NMB0212 | *gyrB* | DNA gyrase subunit B |
| NMB0214 | *prlC* | oligopeptidase A |
| NMB0216 | *katA* | catalase |
| NMB0221 | *pyrD* | dihydroorotate dehydrogenase (dihydroorotate oxidase; DHOdehase; DHODase; DHOD) |
| NMB0232 | *uvrD* | DNA helicase II |
| NMB0243 | *nuoC* | NADH-quinone oxidoreductase chain C (NADH dehydrogenase I, chain C; NDH-1, chain C) |
| NMB0244 | *nuoD* | NADH-quinone oxidoreductase chain D (NADH dehydrogenase I, chain D; NDH-1, chain D) |
| NMB0245 | *nuoE* | NADH-quinone oxidoreductase chain E (NADH dehydrogenase I, chain E; NDH-1, chain E) |
| NMB0246 | *nuoF* | NADH-quinone oxidoreductase chain F (NADH dehydrogenase I, chain F; NDH-1, chain F) |
| NMB0250 | *nuoH* | NADH-quinone oxidoreductase chain H (NADH dehydrogenase I, chain H; NDH-1, chain H) |
| NMB0253 | *nuoJ* | NADH-quinone oxidoreductase chain J (NADH dehydrogenase I, chain J; NDH-1, chain J) |
| NMB0259 | *nuoN* | NADH-quinone oxidoreductase chain N (NADH dehydrogenase I, chain N; NDH-1, chain N) |
| NMB0262 | *xseB* | exodeoxyribonuclease VII small subunit (exonuclease VII small subunit) |
| NMB0264 |  | putative ABC transporter ATP-binding protein |
| NMB0265 | *ruvA* | Holliday junction DNA helicase RuvA |
| NMB0267 |  | putative RlpA-like protein |
| NMB0271 |  | putative methyltransferase |
| NMB0277 |  | putative MviN-like protein |
| NMB0279 |  | putative phosphotransferase |
| NMB0282 |  | putative exoribonuclease II (ribonuclease II; RNase II) |
| NMB0286 |  | conserved hypothetical protein |
| NMB0287 |  | putative ATP-dependent helicase DinG (DNA-damage-inducible protein G) |
| NMB0290 |  | putative HTH-type transcriptional regulator |
| NMB0314 |  | conserved hypothetical protein |
| NMB0316 |  | conserved hypothetical integral membrane protein |
| NMB0317 |  | putative GTP cyclohydrolase I (GTP-CH-I) |
| NMB0318 | *emrA* | multidrug resistance protein A |
| NMB0331 | *coaE* | dephospho-CoA kinase (dephosphocoenzyme A kinase) |
| NMB0332 | *pilD* | type IV prepilin-like proteins leader peptide processing enzyme PilD (prepilin peptidase) [Includes: leader peptidase; N- methyltransferase] |
| NMB0334 | *pgi* | glucose-6-phosphate isomerase 2 (GPI 2; phosphoglucose isomerase 2; PGI 2; phosphohexose isomerase 2; PHI 2) |
| NMB0335 | *dapD* | 2,3,4,5-tetrahydropyridine-2,6-dicarboxylate N-succinyltransferase (tetrahydrodipicolinate N-succinyltransferase; THP succinyltransferase; tetrahydropicolinate succinylase) |
| NMB0337 |  | putative branched-chain amino acid aminotransferase (BCAT) |
| NMB0341 | *tspA* | TspA protein |
| NMB0342 |  | putative intracellular septation protein |
| NMB0347 | *dtd* | D-tyrosyl-tRNA(Tyr) deacylase |
| NMB0348 | *dusA* | tRNA-dihydrouridine synthase A |
| NMB0349 | *gluQ* | putative glutamyl-Q tRNA(Asp) synthetase |
| NMB0351 | *tal* | transaldolase |
| NMB0352 | *kpsF* | arabinose-5-phosphate isomerase |
| NMB0359 | *glnA* | glutamine synthetase (glutamate--ammonia ligase) |
| NMB0360 |  | putative AmpG-like permease |
| NMB0366 |  | hypothetical periplasmic protein |
| NMB0377 |  | conserved hypothetical protein |
| NMB0390 |  | putative glycosyl hydrolase |
| NMB0391 | *pgmB* | beta-phosphoglucomutase (beta-PGM) |
| NMB0392 | *nicB* | quinolinate synthetase B protein (L-aspartate oxidase; LASPO) |
| NMB0394 | *nicA* | quinolinate synthetase A protein |
| NMB0395 |  | conserved hypothetical protein |
| NMB0405 |  | putative Mg chelatase-like protein |
| NMB0406 |  | putative cell division protein FtsN |
| NMB0408 | *uppP* | undecaprenyl-diphosphatase (undecaprenyl pyrophosphate phosphatase; bacitracin resistance protein) |
| NMB0409 |  | conserved hypothetical protein |
| NMB0416 | *murF* | UDP-N-acetylmuramoyl-tripeptide--D-alanyl-D-alanine ligase (UDP-MurNAc-pentapeptide synthetase; D-alanyl-D-alanine-adding enzyme) |
| NMB0420 | *murD* | UDP-N-acetylmuramoylalanine--D-glutamate ligase (UDP-N-acetylmuramoyl-L-alanyl-D-glutamate synthetase; D-glutamic acid adding enzyme) |
| NMB0422 | *murG* | UDP-N-acetylglucosamine--N-acetylmuramyl-(pentapeptide) pyrophosphoryl-undecaprenol N-acetylglucosamine transferase (undecaprenyl-PP-MurNAc-pentapeptide-UDPGlcNAc GlcNAc transferase) |
| NMB0424 | *ddl* | D-alanine--D-alanine ligase (D-alanylalanine synthetase; D-Ala-D-Ala ligase) |
| NMB0434 |  | conserved hypothetical protein |
| NMB0439 |  | putative NnrS-like protein |
| NMB0445 |  | putative transporter |
| NMB0448 | *pdxJ* | pyridoxal phosphate biosynthetic protein PdxJ (PNP synthase) |
| NMB0452 | *acpS* | holo-(acyl-carrier protein) synthase (holo-ACP synthase; 4'-phosphopantetheinyl transferase AcpS) |
| NMB0453 |  | putative mutator MutT protein (7,8-dihydro-8-oxoguanine-triphosphatase; 8-oxo-dGTPase; dGTP pyrophosphohydrolase) |
| NMB0455 |  | putative methylase |
| NMB0457 |  | putative ATPase |
| NMB0458 | *murI* | glutamate racemase |
| NMB0464 |  | putative phospholipase A1 (detergent-resistant phospholipase A; DR-phospholipase A; phosphatidylcholine 1-acylhydrolase; outer-membrane phospholipase A; OM PLA) |
| NMB0465 |  | conserved hypothetical integral membrane protein |
| NMB0472 | *bioF* | 8-amino-7-oxonoanoate synthase (AONS; 8-amino-7-ketopelargonate synthase; 7-keto-8-amino-pelargonic acid synthetase; 7-KAP synthetase; L-alanine--pimelyl CoA ligase) |
| NMB0474 | *bioC* | biotin biosynthesis protein BioC |
| NMB0484 |  | hypothetical protein |
| NMB0485 |  | conserved hypothetical protein |
| NMB0527 |  | putative 6-pyruvoyl tetrahydrobiopterin synthase (PTPS; PTP synthase) |
| NMB0531 |  | putative Na+/H+ antiporter |
| NMB0532 |  | putative Do-like serine protease |
| NMB0536 |  | putative Na(+)/H(+) antiporter |
| NMB0539 | *hemC* | porphobilinogen deaminase (PBG; hydroxymethylbilane synthase; HMBS; pre-uroporphyrinogen synthase) |
| NMB0540 | *aspC* | aspartate aminotransferase (transaminase A; ASPAT) |
| NMB0548 |  | putative membrane fusion protein |
| NMB0549 |  | putative ABC transporter ATP-binding protein |
| NMB0558 |  | hypothetical protein |
| NMB0563 | *apbE* | thiamine biosynthesis lipoprotein ApbE |
| NMB0564 | *nqrF* | Na(+)-translocating NADH-quinone reductase subunit F (Na(+)-translocating NQR subunit F; Na(+)-NQR subunit F; NQR complex subunit F; NQR-1 subunit F) |
| NMB0569 | *nqrA* | Na(+)-translocating NADH-quinone reductase subunit A (Na(+)-translocating NQR subunit A; Na(+)-NQR subunit A; NQR complex subunit A; NQR-1 subunit A) |
| NMB0574 | *gcvT* | glycine cleavage system T protein (aminomethyltransferase) |
| NMB0576 | *hemA* | glutamyl-tRNA reductase (GluTR) |
| NMB0578 |  | truncated NosD-like protein (C-terminal 90% of the protein) |
| NMB0581 |  | putative electron transfer flavoprotein-ubiquinone oxidoreductase (ETF-QO; ETF-ubiquinone oxidoreductase; ETF dehydrogenase; electron-transferring-flavoprotein dehydrogenase) |
| NMB0590 | *trmD* | tRNA (guanine-N(1)-)-methyltransferase (M1G-methyltransferase; tRNA [GM37] methyltransferase) |
| NMB0593 |  | conserved hypothetical protein |
| NMB0596 |  | conserved hypothetical integral membrane protein |
| NMB0600 | *tatB* | sec-independent protein translocase TatB component |
| NMB0607 | *secD* | preprotein translocase SecD subunit |
| NMB0608 | *secF* | preprotein translocase SecF subunit |
| NMB0617 | *rho* | transcription termination factor Rho |
| NMB0618 | *ppsA* | phosphoenolpyruvate synthase (pyruvate, water dikinase; PEP synthase) |
| NMB0620 | *gph* | phosphoglycolate phosphatase 2 (PGPase 2; PGP 2) |
| NMB0621 |  | conserved hypothetical protein |
| NMB0625 |  | conserved hypothetical protein |
| NMB0626 | *prfC* | peptide chain release factor 3 (RF-3) |
| NMB0634 | *fbpA* | iron(III)-transport system iron-binding protein FbpB (major ferric iron-binding protein; FBP; major iron-regulated protein; MIRP; Iron(III) periplasmic binding protein) |
| NMB0637 | *argH* | argininosuccinate lyase (arginosuccinase; ASAL) |
| NMB0666 | *ligA* | DNA ligase (polydeoxyribonucleotide synthase [NAD+]) |
| NMB0669 |  | putative aminodeoxychorismate lyase |
| NMB0670 | *tmk* | thymidylate kinase (dTMP kinase) |
| NMB0671 |  | putative NADP-dependent malic enzyme (NADP-ME) |
| NMB0675 | *kdsB* | 3-deoxy-manno-octulosonate cytidylyltransferase (CMP-KDO synthetase; CMP-2-keto-3-deoxyoctulosonic acid synthetase; CKS) |
| NMB0680 |  | conserved hypothetical periplasmic protein |
| NMB0682 | *pyrC* | dihydroorotase (DHOase) |
| NMB0687 |  | putative Era-like GTP-binding protein |
| NMB0690 | *purF* | amidophosphoribosyltransferase (glutamine phosphoribosylpyrophosphate amidotransferase; ATASE; GPATase) |
| NMB0693 | *folC* | FolC bifunctional protein [includes: folylpolyglutamate synthase (folylpoly-gamma-glutamate synthetase; FPGS; tetrahydrofolate synthase; tetrahydrofolylpolyglutamate synthase) and dihydrofolate synthase] |
| NMB0696 |  | putative ABC transporter ATP-binding protein |
| NMB0698 |  | putative DNA glycosylase |
| NMB0699 | *trpB* | tryptophan synthase beta chain |
| NMB0702 | *comA* | competence protein ComA |
| NMB0703 | *comL* | competence lipoprotein ComL |
| NMB0704 | *rluD* | ribosomal large subunit pseudouridine synthase D (rRNA-uridine isomerase D; rRNA pseudouridylate synthase D) |
| NMB0706 |  | conserved hypothetical protein |
| NMB0711 |  | conserved hypothetical protein |
| NMB0724 | *pheS* | phenylalanyl-tRNA synthetase alpha chain (phenylalanine--tRNA ligase alpha chain; PheRS) |
| NMB0732 | *bioA* | adenosylmethionine-8-amino-7-oxononanoate aminotransferase (7,8-diamino-pelargonic acid aminotransferase; DAPA aminotransferase) |
| NMB0733 | *bioD* | dethiobiotin synthetase (dethiobiotin synthase; DTB synthetase; DTBS) |
| NMB0734 |  | conserved hypothetical protein |
| NMB0736 |  | putative phosphotransferase enzyme IIA component (PTS system EIIA component) |
| NMB0737 | *hprK* | HPr kinase/phosphorylase (HPrK/P; HPr(Ser) kinase/phosphorylase) |
| NMB0738 |  | conserved hypothetical membrane-associated protein |
| NMB0742 |  | conserved hypothetical protein |
| NMB0747 |  | putative rRNA methyltransferase |
| NMB0751 | *xerD* | tyrosine recombinase XerD |
| NMB0758 | *pnp* | polyribonucleotide nucleotidyltransferase (polynucleotide phosphorylase; PNPase; CAP87K) |
| NMB0760 | *dapF* | diaminopimelate epimerase (DAP epimerase) |
| NMB0766 |  | putative GTP-binding protein LepA |
| NMB0767 | *pfs* | MTA/SAH nucleosidase (5'-methylthioadenosine nucleosidase; S-adenosylhomocysteine nucleosidase) |
| NMB0768 | *pilT* | PilT-like protein PilT2 |
| NMB0781 | *hemE* | uroporphyrinogen decarboxylase (URO-D; UPD) |
| NMB0782 | *radA* | DNA repair protein RadA (DNA repair protein Sms) |
| NMB0783 |  | conserved hypothetical periplasmic protein |
| NMB0789 |  | putative amino acid ABC transporter ATP-binding protein |
| NMB0790 | *pgm* | phosphoglucomutase (glucose phosphomutase; PGM) |
| NMB0795 | *pth* | peptidyl-tRNA hydrolase (PTH) |
| NMB0801 | *hemB* | delta-aminolevulinic acid dehydratase (porphobilinogen synthase; ALAD; ALADH) |
| NMB0806 | *rluF* | ribosomal large subunit pseudouridine synthase F (rRNA-uridine isomerase F; rRNA pseudouridylate synthase F) |
| NMB0809 |  | conserved hypothetical protein |
| NMB0814 | *hisZ* | ATP phosphoribosyltransferase regulatory subunit |
| NMB0815 | *purA* | adenylosuccinate synthetase (IMP--aspartate ligase; AdSS; AMPSase) |
| NMB0825 | *rfaE* | D-beta-D-heptose 7-phosphate kinase (D-beta-D-heptose 7-phosphotransferase) |
| NMB0828 | *rfaD* | ADP-L-glycero-D-manno-heptose-6-epimerase (ADP-L-glycero-beta-D-manno-heptose-6-epimerase; ADP-glyceromanno-heptose 6-epimerase; ADP-hep 6-epimerase; AGME) |
| NMB0836 | *clpA* | ATP-dependent Clp protease ATP-binding subunit ClpA |
| NMB0839 |  | putative PmbA-like protein |
| NMB0843 | *pcnB* | poly(A) polymerase (PAP; plasmid copy number protein) |
| NMB0848 |  | conserved hypothetical periplasmic protein |
| NMB0851 | *rdgC* | recombination associated protein RdgC |
| NMB0852 |  | putative GTP-binding protein EngA |
| NMB0854 | *hisS* | histidyl-tRNA synthetase (histidine--tRNA ligase; HisRS) |
| NMB0866 |  | conserved hypothetical outer-membrane protein |
| NMB0868 |  | putative hydrolase |
| NMB0869 | *speE* | spermidine synthase (putrescine aminopropyltransferase; SPDSY) |
| NMB0870 | *panB* | 3-methyl-2-oxobutanoate hydroxymethyltransferase (ketopantoate hydroxymethyltransferase) |
| NMB0871 | *panC* | pantoate--beta-alanine ligase (pantothenate synthetase; pantoate activating enzyme) |
| NMB0872 |  | conserved hypothetical TPR-containing periplasmic protein |
| NMB0874 | *ispE* | 4-diphosphocytidyl-2-C-methyl-D-erythritol kinase (CMK; 4-(cytidine-5'-diphospho)-2-C-methyl-D-erythritol kinase) |
| NMB0876 | *rplY* | 50S ribosomal protein L25 |
| NMB0877 | *dacC* | D-alanyl-D-alanine carboxypeptidase (DD-peptidase; DD-carboxypeptidase; CPase; PBP5) |
| NMB0878 | *ilvA* | threonine dehydratase biosynthetic (threonine deaminase) |
| NMB0879 | *cysA* | sulfate/thiosulfate import ATP-binding protein CysA (sulfate-transporting ATPase) |
| NMB0880 | *cysW* | sulfate transport system permease protein CysW |
| NMB0881 | *cysT* | sulfate transport system permease protein CysT |
| NMB0885 | *dnaB* | replicative DNA helicase |
| NMB0890 | *pilX* | minor pilin PilX |
| NMB0893 | *dut* | deoxyuridine 5'-triphosphate nucleotidohydrolase (dUTPase; dUTP pyrophosphatase) |
| NMB0894 |  | putative aminotransferase |
| NMB0895 |  | conserved hypothetical protein |
| NMB0924 |  | putative oxidoreductase |
| NMB0928 |  | conserved hypothetical lipoprotein |
| NMB0929 | *dapA* | dihydrodipicolinate synthase (DHDPS) |
| NMB0931 | *rlmB* | 23S rRNA (guanosine-2'-O-)-methyltransferase RlmB (23S rRNA Gm2251 2'-O-methyltransferase) |
| NMB0932 |  | conserved hypothetical integral membrane protein |
| NMB0933 | *tadA* | tRNA-specific adenosine deaminase |
| NMB0935 | *miaA* | tRNA delta(2)-isopentenylpyrophosphate transferase (IPP transferase; isopentenyl-diphosphate:tRNA isopentenyltransferase; IPTase; IPPT) |
| NMB0939 |  | putative methionine biosynthesis protein MetW |
| NMB0940 | *metX* | homoserine O-acetyltransferase (homoserine O-trans-acetylase; homoserine transacetylase; HTA) |
| NMB0943 | *metF* | 5,10-methylenetetrahydrofolate reductase |
| NMB0947 | *lpdA* | dihydrolipoyl dehydrogenase (E3 component of pyruvate complex; dihydrolipoamide dehydrogenase) |
| NMB0950 | *sdhA* | succinate dehydrogenase flavoprotein subunit |
| NMB0951 | *sdhB* | succinate dehydrogenase iron-sulfur protein |
| NMB0963 | *psd* | phosphatidylserine decarboxylase proenzyme [contains: phosphatidylserine decarboxylase alpha and beta chains] |
| NMB0964 |  | putative TonB-dependent receptor |
| NMB0966 | *trpG* | anthranilate synthase component II (glutamine amido-transferase) |
| NMB0967 | *trpD* | anthranilate phosphoribosyltransferase |
| NMB0978 | *pntB* | NAD(P) transhydrogenase subunit beta (pyridine nucleotide transhydrogenase subunit beta; nicotinamide nucleotide transhydrogenase subunit beta) |
| NMB0980 | *pntA* | NAD(P) transhydrogenase subunit alpha (pyridine nucleotide transhydrogenase subunit alpha; nicotinamide nucleotide transhydrogenase subunit alpha) |
| NMB0981 | *serB* | phosphoserine phosphatase (PSP; O-phosphoserine phosphohydrolase; PSPase) |
| NMB0997 | *dld* | D-lactate dehydrogenase |
| NMB0999 | *dusC* | tRNA-dihydrouridine synthase C |
| NMB1021 | *trpE* | anthranilate synthase component I (glutamine amido-transferase) |
| NMB1023 |  | putative ATPase |
| NMB1026 |  | conserved hypothetical protein |
| NMB1028 |  | putative transporter |
| NMB1029 | *aspA* | aspartate ammonia-lyase (aspartase) |
| NMB1031 | *leuB* | 3-isopropylmalate dehydrogenase (beta-IPM dehydrogenase; IMDH; 3-IPM-DH) |
| NMB1034 | *leuD* | 3-isopropylmalate dehydratase small subunit (isopropylmalate isomerase; alpha-IPM isomerase; IPMI) |
| NMB1036 | *leuC* | 3-isopropylmalate dehydratase large subunit (isopropylmalate isomerase; alpha-IPM isomerase; IPMI) |
| NMB1037 |  | putative glutamate--cysteine ligase (gamma-glutamylcysteine synthetase) |
| NMB1038 |  | putative DNA repair protein RadC |
| NMB1039 |  | conserved hypothetical protein |
| NMB1041 |  | putative GTPase |
| NMB1044 | *fpr* | ferredoxin--NADP reductase (FNR; flavodoxin reductase; FLXR; FLDR) |
| NMB1045 |  | conserved hypothetical protein |
| NMB1046 | *thrC* | threonine synthase |
| NMB1055 | *glyA* | serine hydroxymethyltransferase (serine methylase; SHMT) |
| NMB1060 | *fbp* | fructose-1,6-bisphosphatase (D-fructose-1,6-bisphosphate 1-phosphohydrolase; FBPase) |
| NMB1061 |  | conserved hypothetical protein |
| NMB1062 |  | conserved hypothetical integral membrane protein |
| NMB1064 | *nudF* | ADP-ribose pyrophosphatase (ADP-ribose diphosphatase; adenosine diphosphoribose pyrophosphatase; ADPR-PPase; ADP-ribose phosphohydrolase; ASPPase) |
| NMB1065 |  | putative CrcB-like protein |
| NMB1068 | *proA* | gamma-glutamyl phosphate reductase (GPR; glutamate-5-semialdehyde dehydrogenase; glutamyl-gamma-semialdehyde dehydrogenase; GSA dehydrogenase) |
| NMB1072 | *lgt* | prolipoprotein diacylglyceryl transferase |
| NMB1073 |  | putative carboxypeptidase |
| NMB1126 |  | putative CsgG-like lipoprotein |
| NMB1128 |  | putative oxidoreductase |
| NMB1133 |  | conserved hypothetical ANK repeat-containing protein |
| NMB1145 | *mpl* | UDP-N-acetylmuramate:L-alanyl-gamma-D-glutamyl-meso-diaminopimelate ligase (murein peptide ligase) |
| NMB1153 | *cysN* | sulfate adenylyltransferase subunit 1 (sulfate adenylate transferase; SAT; ATP-sulfurylase large subunit) |
| NMB1159 |  | conserved hypothetical protein |
| NMB1199 |  | putative GTP-binding protein TypA (tyrosine-phosphorylated protein A) |
| NMB1200 | *rnr* | ribonuclease R (RNase R; VacB protein) |
| NMB1201 | *guaB* | inosine-5'-monophosphate dehydrogenase (IMP dehydrogenase; IMPDH; IMPD) |
| NMB1203 | *glnD* | [protein-PII] uridylyltransferase (PII uridylyl-transferase; uridylyl removing enzyme; UTase) |
| NMB1206 | *bfrB* | bacterioferritin B (BFR B) |
| NMB1218 |  | conserved hypothetical protein |
| NMB1220 |  | conserved hypothetical periplasmic protein |
| NMB1222 | *ung* | uracil-DNA glycosylase (UDG) |
| NMB1226 |  | putative ABC transporter ATP-binding protein |
| NMB1229 |  | hypothetical periplasmic protein |
| NMB1231 | *lon* | ATP-dependent protease Lon |
| NMB1233 | *recD* | exodeoxyribonuclease V alpha chain |
| NMB1234 | *lolD* | lipoprotein-releasing system ATP-binding protein LolD |
| NMB1242 |  | conserved hypothetical protein |
| NMB1243 | *ruvB* | Holliday junction DNA helicase RuvB |
| NMB1250 |  | putative two-component system transcriptional regulator protein |
| NMB1253 |  | conserved hypothetical protein |
| NMB1258 |  | putative ATPase |
| NMB1270 |  | putative methyltransferase |
| NMB1273 |  | putative poly(beta-D-mannuronate) O-acetylase (alginate biosynthesis protein AlgI) |
| NMB1276 | *fadD* | long-chain-fatty-acid--CoA ligase (long-chain acyl-CoA synthetase) |
| NMB1277 |  | putative transporter |
| NMB1280 |  | putative acyl-CoA dehydrogenase |
| NMB1283 | *kdsA* | 2-dehydro-3-deoxyphosphooctonate aldolase (phospho-2-dehydro-3-deoxyoctonate aldolase; 3-deoxy-D-manno-octulosonic acid 8-phosphate synthetase; KDO-8-phosphate synthetase; KDO 8-P synthase; KDOPS) |
| NMB1284 |  | conserved hypothetical integral membrane protein |
| NMB1285 | *eno* | enolase (2-phosphoglycerate dehydratase; 2-phospho-D-glycerate hydro-lyase) |
| NMB1288 | *nrdB* | ribonucleoside-diphosphate reductase beta subunit (ribonucleotide reductase) |
| NMB1291 | *nrdA* | ribonucleoside-diphosphate reductase alpha subunit (ribonucleotide reductase) |
| NMB1301 | *rpsA* | 30S ribosomal protein S1 |
| NMB1304 |  | putative alcohol dehydrogenase class III (S-(hydroxymethyl)glutathione dehydrogenase; glutathione-dependent formaldehyde dehydrogenase; FDH;FALDH) |
| NMB1308 |  | conserved hypothetical protein |
| NMB1310 | *ispG* | 4-hydroxy-3-methylbut-2-en-1-yl diphosphate synthase (1-hydroxy-2-methyl-2-(E)-butenyl 4-diphosphate synthase) |
| NMB1314 | *ftsK* | DNA translocase FtsK |
| NMB1318 | *pss* | CDP-diacylglycerol--serine O-phosphatidyltransferase (phosphatidylserine synthase) |
| NMB1319 |  | conserved hypothetical integral membrane protein |
| NMB1328 | *trmB* | tRNA (guanine-N(7)-)-methyltransferase (tRNA(m7G46)-methyltransferase) |
| NMB1338 |  | putative hydrolase |
| NMB1339 | *proS* | prolyl-tRNA synthetase (proline--tRNA ligase; ProRS; global RNA synthesis factor) |
| NMB1341 | *aceE* | pyruvate dehydrogenase E1 component |
| NMB1347 | *suhB* | inositol-1-monophosphatase (IMPase; inositol-1-phosphatase; I-1-Pase) |
| NMB1354 |  | putative transporter |
| NMB1362 |  | putative transporter |
| NMB1365 |  | conserved hypothetical protein |
| NMB1371 | *argD* | acetylornithine/succinyldiaminopimelate aminotransferase (ACOAT; succinyldiaminopimelate transferase; DapATase) |
| NMB1372 | *clpX* | ATP-dependent Clp protease ATP-binding subunit ClpX |
| NMB1373 | *rbfA* | ribosome-binding factor A |
| NMB1377 | *lldA* | L-lactate dehydrogenase (cytochrome) |
| NMB1388 | *pgi* | glucose-6-phosphate isomerase 1 (GPI 1; phosphoglucose isomerase 1; PGI 1; phosphohexose isomerase 1; PHI 1) |
| NMB1392 | *zwf* | glucose-6-phosphate 1-dehydrogenase (G6PD) |
| NMB1396 | *mutY* | A/G-specific adenine glycosylase |
| NMB1417 |  | putative polynucleotidyl transferase |
| NMB1418 |  | putative lipid A biosynthesis (KDO)2-(lauroyl)-lipid IVA acyltransferase |
| NMB1419 | *ruvC* | crossover junction endodeoxyribonuclease RuvC (Holliday junction nuclease RuvC; Holliday junction resolvase RuvC) |
| NMB1420 |  | putative Fis-like DNA-binding protein |
| NMB1428 |  | putative metallopeptidase |
| NMB1429 | *porA* | major outer-membrane protein P,IA (protein IA; PIA; porin) |
| NMB1437 |  | conserved hypothetical protein |
| NMB1443 | *dnaZX* | DNA polymerase III tau/gamma subunits |
| NMB1445 | *recA* | RecA protein (recombinase A) |
| NMB1446 | *aroD* | 3-dehydroquinate dehydratase (3-dehydroquinase; type I DHQase) |
| NMB1452 |  | putative dioxygenase (pseudogene part 1) |
| NMB1457 | *tkt* | transketolase (TK) |
| NMB1458 | *fumC* | fumarate hydratase class II (fumarase C) |
| NMB1461 |  | putative transporter |
| NMB1471 | *trpS* | tryptophanyl-tRNA synthetase (tryptophan--tRNA ligase; TrpRS) |
| NMB1473 |  | putative aminotransferase |
| NMB1476 | *gdhB* | NAD-specific glutamate dehydrogenase (NAD-GDH) |
| NMB1483 |  | putative metallopeptidase |
| NMB1487 |  | conserved hypothetical protein |
| NMB1488 | *gabD* | succinate-semialdehyde dehydrogenase [NADP+] (SSDH) |
| NMB1497 |  | putative TonB-dependent receptor |
| NMB1501 |  | conserved hypothetical protein |
| NMB1505 | *pncB* | nicotinate phosphoribosyltransferase (NAPRTase) |
| NMB1506 | *argS* | arginyl-tRNA synthetase (arginine--tRNA ligase; ArgRS) |
| NMB1508 |  | conserved hypothetical integral membrane protein |
| NMB1513 | *ispD* | 2-C-methyl-D-erythritol 4-phosphate cytidylyltransferase (4-diphosphocytidyl-2C-methyl-D-erythritol synthase; MEP cytidylyltransferase; MCT) |
| NMB1514 | *dnaQ* | DNA polymerase III epsilon subunit |
| NMB1524 |  | putative D-lactate dehydrogenase [cytochrome] (D-lactate ferricytochrome c oxidoreductase; D-LCR) |
| NMB1527 | *rfaF* | ADP-heptose:LPS heptosyltransferase II |
| NMB1528 |  | methylated-DNA--protein-cysteine methyltransferase (6-O-methylguanine-DNA methyltransferase; MGMT; O-6-methylguanine-DNA-alkyltransferase) |
| NMB1530 | *dapE* | succinyl-diaminopimelate desuccinylase (SDAP) |
| NMB1531 |  | conserved hypothetical protein |
| NMB1533 |  | H,8 outer-membrane lipoprotein |
| NMB1554 | *pyrG* | CTP synthase (UTP--ammonia ligase; CTP synthetase) |
| NMB1556 | *trmU* | tRNA (5-methylaminomethyl-2-thiouridylate)-methyltransferase |
| NMB1559 | *gshB* | glutathione synthetase (glutathione synthase; GSH synthetase; GSH-S; GSHase) |
| NMB1561 |  | putative HTH-type transcriptional regulator |
| NMB1564 |  | putative OsmC-like protein |
| NMB1572 | *acnB* | aconitate hydratase 2 (citrate hydro-lyase 2; aconitase 2) |
| NMB1573 | *argF* | ornithine carbamoyltransferase (OTCase) |
| NMB1577 | *ilvI* | acetolactate synthase large subunit (AHAS; acetohydroxy-acid synthase large subunit; ALS) |
| NMB1586 |  | putative transporter |
| NMB1588 | *pgsA* | CDP-diacylglycerol--glycerol-3-phosphate 3-phosphatidyltransferase (phosphatidylglycerophosphate synthase; PGP synthase) |
| NMB1590 |  | conserved hypothetical protein |
| NMB1605 | *parC* | topoisomerase IV subunit A |
| NMB1608 |  | conserved hypothetical protein |
| NMB1613 | *fumA* | fumarate hydratase class I (fumarase) |
| NMB1616 | *thiD* | phosphomethylpyrimidine kinase (HMP-phosphate kinase; HMP-P kinase) |
| NMB1619 |  | conserved hypothetical protein |
| NMB1620 |  | putative muramoyltetrapeptide carboxypeptidase (LD-carboxypeptidase A) |
| NMB1640 | *serC* | phosphoserine aminotransferase (PSAT) |
| NMB1642 | *nusA* | transcription elongation protein nusA (N utilization substance protein A; L factor) |
| NMB1645 |  | conserved hypothetical integral membrane protein |
| NMB1652 |  | conserved hypothetical protein |
| NMB1654 |  | conserved hypothetical periplasmic protein |
| NMB1658 | *coaBC* | coenzyme A biosynthesis bifunctional protein CoaBC (DNA/pantothenate metabolism flavoprotein) [includes: phosphopantothenoylcysteine decarboxylase (PPCDC; CoaC) and phosphopantothenate--cysteine ligase (phosphopantothenoylcysteine synthase; PPC synthetase |
| NMB1659 | *spoT* | guanosine-3',5'-bis(diphosphate) 3'-pyrophophohydrolase ((ppGpp)ase; penta-phosphate guanosine-3'-pyrophosphohydrolase) |
| NMB1664 |  | putative peptidase |
| NMB1669 | *hemO* | heme oxygenase HemO |
| NMB1672 |  | conserved hypothetical lipoprotein |
| NMB1673 | *tag* | DNA-3-methyladenine glycosylase I (3-methyladenine-DNA glycosylase I, constitutive; TAG I; DNA-3-methyladenine glycosidase I) |
| NMB1674 |  | putative lipolytic enzyme |
| NMB1675 |  | hypothetical integral membrane protein |
| NMB1678 | *tyrB* | aromatic amino acid aminotransferase (AROAT; ARAT) |
| NMB1679 | *trmA* | tRNA (uracil-5-)-methyltransferase (tRNA(M-5-U54)-methyltransferase; RUMT) |
| NMB1680 | *aroC* | chorismate synthase (5-enolpyruvylshikimate-3-phosphate phospholyase) |
| NMB1682 | *parE* | topoisomerase IV subunit B |
| NMB1684 | *serS* | seryl-tRNA synthetase (serine--tRNA ligase; SerRS) |
| NMB1685 | *ldhA* | D-lactate dehydrogenase (D-LDH; fermentative lactate dehydrogenase) |
| NMB1686 | *prfA* | peptide chain release factor I (RF-1) |
| NMB1693 |  | putative AsmA-like protein |
| NMB1694 | *ubiD* | 3-octaprenyl-4-hydroxybenzoate carboxy-lyase (polyprenyl p-hydroxybenzoate decarboxylase) |
| NMB1707 |  | putative sodium-dependent transporter |
| NMB1711 |  | putative HTH-type transcriptional regulator |
| NMB1715 | *mtrD* | drug efflux transport protein MtrD |
| NMB1716 | *mtrC* | membrane fusion protein MtrC |
| NMB1735 | *relA* | GTP pyrophosphokinase (ATP:GTP 3'-pyrophosphotransferase; ppGpp synthetase I; (P)ppGpp synthetase) |
| NMB1743 |  | hypothetical protein |
| NMB1780 | *tpsB* | TpsA3 activation/secretion protein TpsB3 |
| NMB1787 | *argC* | N-acetyl-gamma-glutamyl-phosphate reductase (AGPR; N-acetyl-glutamate semialdehyde dehydrogenase; NAGSA dehydrogenase) |
| NMB1788 | *recG* | ATP-dependent DNA helicase RecG |
| NMB1799 | *metK* | S-adenosylmethionine synthetase (methionine adenosyltransferase; AdoMet synthetase; MAT) |
| NMB1801 |  | putative lipid A biosynthesis lauroyl acyltransferase (heat shock protein B) |
| NMB1804 |  | putative cytochrome c biogenesis protein |
| NMB1817 | *ribD* | riboflavin biosynthesis protein RibD [includes: diaminohydroxyphosphoribosylaminopyrimidine deaminase (riboflavin-specific deaminase) and 5-amino-6-(5-phosphoribosylamino)uracil reductase (HTP reductase)] |
| NMB1822 | *pglD* | pilin glycosylation protein PglD |
| NMB1834 | *ribF* | riboflavin biosynthesis protein RibF [includes: riboflavin kinase (flavokinase) and FMN adenylyltransferase (FAD pyrophosphorylase; FAD synthetase)] |
| NMB1835 | *tyrS* | tyrosyl-tRNA synthetase (tyrosine--tRNA ligase; TyrRS) |
| NMB1838 |  | putative GTP-dependent nucleic acid-binding protein EngD |
| NMB1839 | *fhs* | formate--tetrahydrofolate ligase (formyltetrahydrofolate synthetase; FHS; FTHFS) |
| NMB1840 |  | conserved hypothetical integral membrane protein |
| NMB1846 |  | putative ATP-binding protein |
| NMB1855 | *carB* | carbamoyl-phosphate synthase large chain (carbamoyl-phosphate synthetase ammonia chain) |
| NMB1859 | *queA* | S-adenosylmethionine:tRNA ribosyltransferase-isomerase (queuosine biosynthesis protein QueA) |
| NMB1861 | *accC* | biotin carboxylase (A subunit of acetyl-CoA carboxylase; ACC) |
| NMB1864 | *hemL* | glutamate-1-semialdehyde 2,1-aminomutase (GSA; glutamate-1-semialdehyde aminotransferase; GSA-AT) |
| NMB1866 |  | putative MiaB-like tRNA modification enzyme |
| NMB1867 | *dxs* | 1-deoxy-D-xylulose-5-phosphate synthase (1-deoxyxylulose-5-phosphate synthase; DXP synthase; DXPS) |
| NMB1868 | *xerC* | tyrosine recombinase XerC |
| NMB1869 | *fba* | fructose-bisphosphate aldolase |
| NMB1871 |  | putative peptidase |
| NMB1872 |  | putative acetyltransferase |
| NMB1873 |  | putative DNA glycosylase |
| NMB1882 |  | putative tonB-dependent ferric siderophore receptor protein |
| NMB1885 | *pcm* | protein-L-isoaspartate O-methyltransferase (protein-beta-aspartate methyltransferase; PIMT; protein L-isoaspartyl methyltransferase; L-isoaspartyl protein carboxyl methyltransferase) |
| NMB1898 |  | conserved hypothetical lipoprotein |
| NMB1899 |  | hypothetical periplasmic protein |
| NMB1907 | *oxaA* | inner-membrane protein OxaA |
| NMB1913 | *plsX* | fatty acid/phospholipid synthesis protein PlsX |
| NMB1916 | *fabH* | 3-oxoacyl-[acyl-carrier-protein] synthase III (beta-ketoacyl-ACP synthase III; KAS III) |
| NMB1918 | *fabD* | malonyl CoA-acyl carrier protein transacylase (MCT) |
| NMB1920 | *guaA* | GMP synthase [glutamine-hydrolyzing] (glutamine amidotransferase; GMP synthetase) |
| NMB1928 | *lgtB* | lacto-N-neotetraose biosynthesis glycosyl transferase LgtB |
| NMB1934 | *atpD* | ATP synthase beta chain |
| NMB1936 | *atpA* | ATP synthase alpha chain |
| NMB1944 |  | putative chromosome partitioning protein ParB |
| NMB1948 |  | putative D-methionine transport system ATP-binding protein MetN |
| NMB1953 |  | putative SspA-like protein |
| NMB1964 |  | conserved hypothetical integral membrane protein |
| NMB1965 |  | conserved hypothetical membrane protein |
| NMB1967 |  | putative HTH-type transcriptional regulator |
| NMB1968 | *aldA* | aldehyde dehydrogenase A (lactaldehyde dehydrogenase) |
| NMB1972 | *groL* | 60 kDa chaperonin (protein Cpn60; GroEL protein; 63 kDa stress protein; GSP63; HSP60) |
| NMB1980 |  | conserved hypothetical protein |
| NMB1981 | *luxS* | S-ribosylhomocysteine lyase (autoinducer-2 production protein LuxS; AI-2 synthesis protein) |
| NMB1985 | *app* | autotransporter App |
| NMB1987 | *trmE* | tRNA modification GTPase TrmE |
| NMB1989 | *fetB* | enterobactin uptake system binding lipoprotein FetB |
| NMB1992 |  | conserved hypothetical protein |
| NMB1993 |  | putative ferric enterobactin uptake system ATP-binding protein FetE |
| NMB1997 |  | putative hydroxyacylglutathione hydrolase (glyoxalase II; Glx II) |
| NMB2000 | *hslO* | 33 kDa chaperonin (heat shock protein 33 homolog; HSP33) |
| NMB2024 |  | putative nicotinate-nucleotide adenylyltransferase (deamido-NAD(+) pyrophosphorylase; deamido-NAD(+) diphosphorylase; nicotinate mononucleotide adenylyltransferase; NaMN adenylyltransferase) |
| NMB2029 | *thrB* | homoserine kinase (HSK; HK) |
| NMB2030 | *ubiG* | 3-demethylubiquinone-9 3-methyltransferase (3,4-dihydroxy-5-hexaprenylbenzoate methyltransferase; DHHB methyltransferase) |
| NMB2031 |  | putative aromatic amino acid transporter |
| NMB2033 | *gmhB* | D,D-heptose 1,7-bisphosphate phosphatase (D-glycero-D-manno-heptose 1,7-bisphosphate phosphatase) |
| NMB2034 | *nlaB* | 1-acyl-sn-glycerol-3-phosphate acyltransferase NlaB (1-AGP acyltransferase; 1-AGPAT; lysophosphatidic acid acyltransferase; LPAAT) |
| NMB2042 |  | putative ABC transporter ATP-binding protein |
| NMB2044 | *ptsI* | phosphoenolpyruvate-protein phosphotransferase (phosphotransferase system, enzyme I; protein I) |
